# Evolutionary Principles of Bacterial Signaling Capacity and Complexity

**DOI:** 10.1128/mbio.00764-22

**Published:** 2022-05-10

**Authors:** Ran Mo, Yugeng Liu, Yuanyuan Chen, Yingjin Mao, Beile Gao

**Affiliations:** a CAS Key Laboratory of Tropical Marine Bio Resources and Ecology, Guangdong Key Laboratory of Marine Materia Medica, Innovation Academy of South China Sea Ecology and Environmental Engineering, South China Sea Institute of Oceanology, Chinese Academy of Sciences, Guangzhou, China; b Southern Marine Science and Engineering Guangdong Laboratory, Guangzhou, China; c Tropical Marine Biological Research Station in Hainan, Sanya Institute of Oceanology, Chinese Academy of Sciences and Hainan Key Laboratory of Tropical Marine Biotechnology, Sanya, China; d University of Chinese Academy of Sciences, Beijing, China; Iowa State University

**Keywords:** chemotaxis, c-di-GMP, *Campylobacter*, *Helicobacter*

## Abstract

Microbes rely on signal transduction systems to sense and respond to environmental changes for survival and reproduction. It is generally known that niche adaptation plays an important role in shaping the signaling repertoire. However, the evolution of bacterial signaling capacity lacks systematic studies with a temporal direction. In particular, it is unclear how complexity evolved from simplicity or vice versa for signaling networks. Here, we examine the evolutionary processes of major signal transduction systems in *Campylobacterota* (formerly *Epsilonproteobacteria*), a phylum with sufficient evolutionary depth and ecological diversity. We discovered that chemosensory system increases complexity by horizontal gene transfer (HGT) of entire chemosensory classes, and different chemosensory classes rarely mix their components. Two-component system gains complexity by atypical histidine kinases fused with receiver domain to achieve multistep or branched signal transduction process. The presence and complexity of c-di-GMP-mediated system is related to the size of signaling network, and c-di-GMP pathways are easy to rewire, since enzymes and effectors can be linked without direct protein-protein interaction. Overall, signaling capacity and complexity rise and drop together in *Campylobacterota*, determined by sensory demand, genetic resources, and coevolution within the genomic context. These findings reflect plausible evolutionary principles for other cellular networks and genome evolution of the *Bacteria* domain.

## INTRODUCTION

Five decades of vigorous studies have elucidated major signal transduction systems in bacteria, and they came into the limelight in the following order: (i) hemosensory system (also called the chemotaxis system), mostly involved in navigation of motility (1); (ii) two-component system (TCS), primarily for regulation of gene expression (2, 3); (iii) secondary messenger-dependent systems, represented by cyclic adenosine monophosphate (cAMP) (4), cyclic diguanylate (c-di-GMP) (5), cyclic diadenylate (c-di-AMP) (6), and more (7, 8); (iv) serine/threonine/tyrosine protein kinases (STYKs) and protein phosphatase-mediated pathways (9); (v) extracytoplasmic function σ factors (ECFs) as alternative σ factors in redirection of transcription (10); and (vi) quorum sensing (QS) in cell-to-cell communication (11).

There are also one-component systems (OCSs), each composed of a single protein that works by domain-domain interaction (12), but OCS proteins are often assigned to other systems if they contain receiver (REC) domain, GGDEF domain, or other transmitter domains that can generate links with other signaling proteins. While each signal transduction system may stand on its own, they are also intertwined to form a network that confers fitness advantages for bacterial adaptation to the ever-changing environment. Previous census studies have counted and sorted the components in each system across diverse bacterial lineages, learning their system composition and diversity, modularity and plasticity, phylogenetic distribution, correlation with genome size and ecology, and so on (13–19). However, beyond the single-system level, how the signaling capacity of bacterial species evolved remains largely elusive. This general question can be specified as the following questions. (i) How are new components and network links added to increase system complexity? (ii) What kind of genetic innovations accompany the increase in the number of components? (iii) Why are some systems abundant but some are absent from different bacterial species? Answers to these questions are fundamental to understanding the organization of any biological network and also to shed light on the evolution of genome that encodes a supernetwork of all cellular processes.

A systematic approach to tackle these questions would be to track the evolution of the major signal transduction systems in the *Bacteria* based on the species/lineage branching order. Analyses of a large number of diverse bacterial species with finely spaced phylogenetic progression are needed for a full coverage of signaling capacity at different complexity levels. In addition, it is believed that ecological adaptation is the driving force for signaling network evolution, thus species habitat should be considered when comparing the dynamics of the signaling proteins in different species (20). Currently, it is not realistic to analyze the entire *Bacteria* domain, with its enormous number of sequenced genomes, simply because there is no consensus on the root of the bacterial tree and the order of phylum divergence (21, 22). In addition, since these questions focus on the evolutionary processes rather than the origin of signal transduction systems, a starting point is needed but not necessarily the one tracing back to the last bacterial common ancestor. Instead, a monophyletic group with a deep root, well-supported phylogeny, and wide ecological distribution would provide a good representation. We propose that *Campylobacterota* (phyl. nov.), previously *Epsilonproteobacteria* class, is a good model for such a pilot study (23).

## RESULTS

### An eco-evo framework from deep sea to human gut.

**FIG 1:**
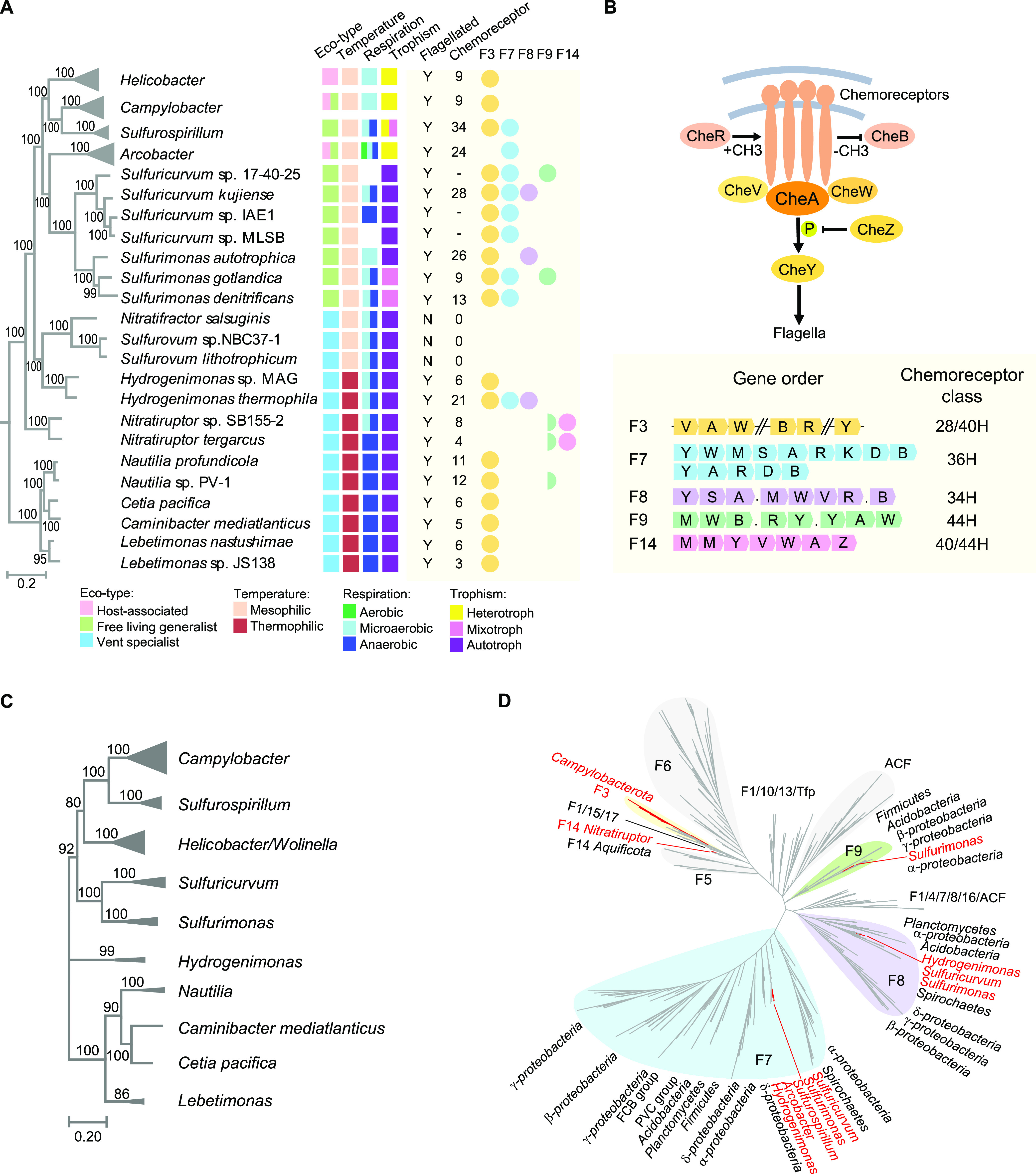
Chemosensory system in *Campylobacterota*. (A) Phylogeny (left), ecophysiological characteristics (middle), and chemosensory system (right) of the *Campylobacterota*. Tree topology is based on Fig. S1 and collapsed for 4 genera with multiple sequenced species but the same chemosensory F class pattern. A hyphen in the chemoreceptor column indicates that the total number of chemoreceptor genes is unavailable due to genome incompleteness; the numbers for 4 collapsed genera on the top are average numbers of chemoreceptors of all representative species of one genus. Full circle on the right represents that the full gene set of specific F class is present; half circle means only half of components of the F class are present. (B) Model of chemosensory system and characteristics of F3/F7/F8/F9/F14 classes in terms of composition, gene order, and chemoreceptor type. Abbreviations in gene order: A, *cheA*; B, *cheB*; D, *cheD*; K, a gene encoding histidine kinase; M, chemoreceptor; R, *cheR*; S, a gene encoding STAS domain alone; V, *cheV*; W, *cheW*; Y, *cheY*. A dot represents hypothetical proteins. (C) Phylogeny of F3 class core proteins (CheA, CheV, and CheW) in *Campylobacterota*. (D) Phylogeny of CheA homologs of distinct F classes from diverse bacterial phyla. The *Campylobacterota* phylum and its genera are highlighted in red.

*Campylobacterota* is one of the phyla with many complete genomes of diverse species based on current NCBI and GTDB taxonomy and genome collections (24, 25). Other bacterial phyla with more than 50 completely sequenced species are either not monophyletic or lack ecological diversity and thus were not selected for this study (see Table S1 at https://data.mendeley.com/datasets/wxwcjzm9wv/1). Both 16S rRNA trees and phylogenomic analyses strongly suggest that the ancestral lineage of this phylum was composed of specialist organisms residing at the deep-sea hydrothermal vents, which are a model system for studying the Archaean Earth environment (23, 26). We reconstructed the phylogeny for all species with completely sequenced genomes from *Campylobacterota* and collected their ecological and physiological details (Fig. 1A; also see Fig. S2 in the supplemental material and see Table S1 at https://data.mendeley.com/datasets/wxwcjzm9wv/1). The deepest branch of this phylum consists of strictly anaerobic and thermophilic chemolithoautotrophs that were isolated only from the deep-sea hydrothermal vents. Their metabolic characteristics and prevalence in the vent microbial community suggest that the ancient *Campylobacterota* played an important role in early Earth history (26). Later lineages underwent niche expansion toward the surface of the ocean and terrestrial water reservoirs; these lineages can be grouped as free-living generalists. Along with ecological diversification, they display physiological transitions from anaerobic to microaerobic/aerobic environment, thermophily to mesophily, and autotrophy to heterotrophy. Furthermore, some lineages became host-associated commensals or pathogens represented by two genera, Campylobacter and *Helicobacter*, obligately mesophilic and heterotrophic. Eventually, some species became restricted to specific hosts, such as Helicobacter pylor and other successful colonizers of human gut.

10.1128/mbio.00764-22.1 FIG S1 Phylogenetic tree and species ecophysiological characteristics of the *Campylobacterota* phylum. Detail information of all species in Table S2 at https://data.mendeley.com/datasets/wxwcjzm9wv/1. Download FIG S1, PDF file, 0.3 MB. Copyright © 2022 Mo et al. 2022 Mo et al. https://creativecommons.org/licenses/by/4.0/ This content is distributed under the terms of the Creative Commons Attribution 4.0 International license.

10.1128/mbio.00764-22.2 FIG S2 Chemosensory classes of *Campylobacterota* species are illustrated in linearized genomes. The color codes of chemosensory classes are noted in the bottom panel; C, cheC; X, cheX; Z, cheZ. Other gene abbreviations are the same as those in Fig. 1B. Download FIG S2, PDF file, 0.5 MB. Copyright © 2022 Mo et al. 2022 Mo et al. https://creativecommons.org/licenses/by/4.0/ This content is distributed under the terms of the Creative Commons Attribution 4.0 International license.

It is clear that *Campylobacterota* has a deep evolutionary origin and a traceable niche expansion from deep-sea hydrothermal vents to diverse habitats of modern lineages, making this phylum suitable for inferring evolutionary processes by assessment of its contemporary diversity. Moreover, species of this phylum have a narrow range of genome size variation (1.4 to ~3.5 Mb) without dramatic genome reduction or expansion largely due to random genetic drift (see Table S2 at https://data.mendeley.com/datasets/wxwcjzm9wv/1). This enables examination of ecological adaptive selection on genome evolution in a stepwise manner. Here, based on this eco-evo framework, we dissect each signal transduction system by following their compositional changes, compare their evolutionary processes, and identify their determinants in the signaling network.

### Chemosensory system: add, reduce, but rarely stir.

The chemosensory system is composed of multicomponent pathways that sense environmental stimuli by different chemoreceptors and integrate all the signals to affect the activity of CheA kinase (27). Activated CheA can autophosphorylate and pass on the phosphoryl group to its response regulator CheY to control motility, biofilm formation, development, or other cellular processes (Fig. 1B). The ultimate complexity of this system is reflected by the presence of several chemosensory pathways/arrays with different composition and structure in some bacterial cells (28, 29). A milestone classification scheme for chemosensory system has sorted them into 19 classes based on gene order, domain architecture, auxiliary component(s), and chemoreceptor types (30). These classes include one type IV pilus class (Tfp), one alternative cellular functions (ACF) class, and 17 flagellar classes (F1 to F17) (30). We followed this classification scheme to analyze all chemosensory genes in *Campylobacterota* species and identified 5 classes (F3/F7/F8/F9/F14) in this phylum. Their distinctive gene order/components within chemosensory gene clusters and corresponding chemoreceptor types are summarized in Fig. 1B.

Mapping all identified chemosensory classes in the eco-evo framework of *Campylobacterota*, we found that F3 class is conserved throughout *Campylobacterota* from hydrothermal vent specialists to host-associated species, except those that are either unflagellated or have distinctive flagellar gene sets (Fig. 1A and Fig. S2). The F3 class is defined by the presence of a core gene operon, *cheVAW*, additional receiver (REC) domain in CheA kinase, and an auxiliary CheB lacking the REC domain (30) (Fig. 1B). A phylogenetic tree based on core proteins of F3 class shows the same branch pattern as the species tree, suggesting that F3 class evolved in the common ancestor of *Campylobacterota* and was vertically passed on to its descendants (Fig. 1C).

In addition to F3, other chemosensory classes are found in free-living generalists, including F7, F8, and F9, coupled with a larger number of chemoreceptor genes (Fig. 1A). These additional F classes likely have been acquired by horizontal gene transfer (HGT), since they branch with other bacterial phyla with the same F class in phylogenetic tree analysis (Fig. 1D). Consistent with this, these classes have all or most of their components encoded by a single cluster, including their cognate chemoreceptors, facilitating genetic transfers within this phylum and from other bacterial phyla of the same habitat (Fig. S2). Moreover, the narrow or sporadic species distribution patterns of these non-F3 classes suggest that they were introduced at different branching points of *Campylobacterota* lineages and subsequently were lost in some species or transferred again within this phylum (Fig. 1A and D). Thus, in contrast to the vertically inherited F3 class, they are easily gained and lost by *Campylobacterota* during niche expansion and completely absent from recently derived lineages associated with animal or human hosts.

To increase the sensing ability for niche expansion, bacteria could simply gain more chemoreceptors by gene duplication/divergence and HGT but use the same transmitter gene sets. However, most free-living generalists examined here acquired extra chemosensory classes, adding complexity to signal transmission while expanding the sensory repertoire. Later, when the lineages became niche specialized, they only retained one F class with a reduced number of chemoreceptors or lost the entire chemosensory system if their flagellar genes were lost. For example, species of the *Sulfurovum* genus that are either ubiquitous in deep-sea vent biofilm or living as epibiotic symbionts of deep-sea animals do not have any chemosensory or flagellar genes, consistent with their sessile lifestyle (Fig. 1A) (31). These results suggest that the presence and complexity of chemosensory system is determined by sensory demand and signal output, such as that of flagella. However, an F chemosensory class does not necessarily control flagellar motility, and many signal outputs for species with multiple chemosensory classes are currently unknown (28). Future functional characterization of all signal outputs in species with complex chemosensory systems will reveal specific determinants for each chemosensory class.

The above-described phylogenetic analyses suggest that all the non-F3 classes in *Campylobacterota* are obtained by HGT and not by duplication and divergence of the ancestral F3 class (Fig. 1D). Moreover, although different chemosensory classes within the same genome undergo dynamic gain and loss, their main transmitter genes rarely mix and match. In all the chemosensory classes of *Campylobacterota*, we only see two examples of mixed F class genes: F3-*cheZY* incorporated into the new F7 class in *Arcobacter* and half the components of F9 class, including chemoreceptor, CheW, CheB, and CheR without its own kinase and response regulator, which must function with the only F class in the species. The fact that only 19 chemosensory classes could be identified among the great diversity of the *Bacteria* domain also indicates that genetic innovation of new chemosensory class is rare during bacterial evolution, and species with multiple chemosensory classes mainly gain extra transmitter sets by HGT. Thus, we conclude that the chemosensory system follows the “add, reduce, but rarely stir” evolutionary mode compared to a description of genome evolution of bacterial pathogens: “add, stir, and reduce” (32). Importantly, the intrinsic “rarely stir” feature ensures a traceable history, making the F class classification scheme work.

### TCS gets complicated with more and more REC domains.

**FIG 2:**
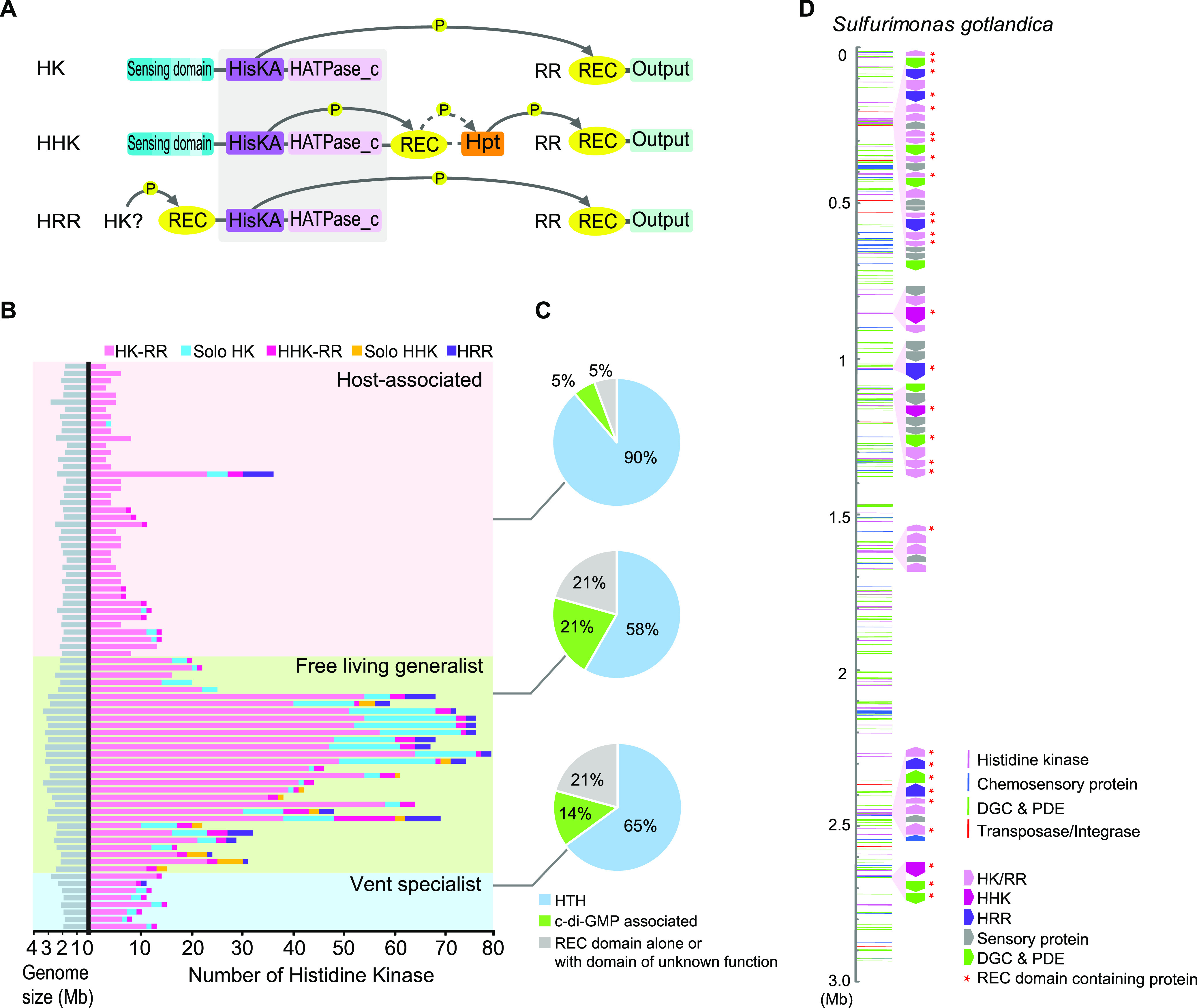
TCSs in *Campylobacterota*. (A) Schematic modular organization of TCS proteins. Phosphotransfer is depicted by solid arrow line and phosphorelay by dashed arrow line. REC, receiver domain; HisKA, histidine kinase phosphoacceptor domain; HATPase_c, histidine kinase-like ATPase domain; Hpt, histidine-containing phosphotransfer domain. (B) The abundance of HKs in *Campylobacterota* species. Details are in Table S3 at https://data.mendeley.com/datasets/wxwcjzm9wv/1. (C) The proportions of output types of RRs in vent specialists, generalists, and host-associated species of *Campylobacterota*. (D) Atypical HK enriched gene clusters in the genome of Sulfurimonas gotlandica GD1, which is linearized for display purposes. All signal transduction genes are represented as colored stripes based on their starting location, and atypical HK enriched regions are labeled beside the genome.

The TCS is typically composed of a sensory histidine kinase (HK) and a response regulator (RR) (33). An HK protein generally has one or more sensor domain(s) at the N terminus and HisKA plus HATPase domains at the C terminus that can autophosphorylate upon activation by the sensor domain and then transfer the phosphoryl group to the REC domain of its cognate RR (Fig. 2A). These HK-RR pairs can hardly make a sophisticated network by increasing their numbers; in fact, few atypical HKs truly expand the diversity and complexity of TCS (34). Based on domain architecture, atypical HKs are classified into two types. One type is hybrid histidine kinase (HHK) with C-terminal REC domain that can perform a phosphorelay or tune its own kinase activity. Another type is hybrid response regulator (HRR) with N-terminal REC domain fused to transmitter domains HisKA and HATPase; importantly, the N-terminal REC domain is phosphorylated by another HK protein rather than its own kinase domain (Fig. 2A) (35, 36). Because of the built-in REC domain, both HHKs and HRRs can participate in multicomponent or multistep signal transduction processes, but it is difficult to delineate their information flow, in contrast to the paired HK-RR paradigm (37, 38).

To evaluate the abundance of TCS in *Campylobacterota* genomes, we counted all HK proteins with both HisKA and HATPase domains and found that the numbers from each species roughly correlate with their ecological breadth (Fig. 2B). Both vent specialists and host-associated groups have relatively fewer and typically classical TCS pairs (~10 or less), while free-living generalists have fair amounts of solo HKs, HHKs, and HRRs in addition to the paired HKs, with a peak number of 79 HKs in total. Accordingly, the number of RRs increases with that of HKs, and their output domains also diversify. Besides the RRs with the DNA-binding helix-turn-helix domain as the common output to regulate gene expression, 21% of RRs in free-living *Campylobacterota* generalists have c-di-GMP turnover enzymes as signal outputs and another 21% have other domains of mostly unknown function (Fig. 2C). As previously reported, TCS can easily integrate with other signaling systems through the fusion of REC domain with noncanonical modules (36, 39).

These analyses highlighted two notable trends for HHKs and HRRs: their amount is positively related to the total number of HKs, and the genes encoding them are frequently flanked by TCS gene pairs, standalone sensor domain genes, and c-di-GMP turnover genes (Fig. 2B and Fig. S3). For example, the complexity apex of TCSs in *Campylobacterota* is represented by Sulfurimonas gotlandica GD1, with the total number of solo HKs, HHKs, and HRRs in combination reaching 45% of all HKs (Fig. 2B; see also Table S3 at https://data.mendeley.com/datasets/wxwcjzm9wv/1). This genome has 10 clusters enriched in atypical HKs; the biggest cluster contains 22 signaling proteins (Fig. 2D and Fig. S4). Functional relatedness suggests that proteins encoded by the same gene cluster participate in the same pathway(s) with several sensors and checkpoints, organized as a branched and multistep phosphorelay to elicit a specific output response. Moreover, REC domain is the most recurrent module in these clusters, either fused with other modules to link different transmitters or standing alone as the phosphate sink to fine-tune the signaling process (Fig. S4).

10.1128/mbio.00764-22.3 FIG S3 Domain architectures of all HKs and RRs in representative species of each genus within the *Campylobacterota* phylum. HHKs are highlighted in blue, and HRRs are highlighted in red. The solid line connects the adjacent HK and RR genes; dashed line represents potential 1:1 functional link of HK and RR gene pair within four genes distance; dotted line indicates potential functional links among multiple HK and RR genes within four genes distance. Download FIG S3, PDF file, 2.7 MB. Copyright © 2022 Mo et al. 2022 Mo et al. https://creativecommons.org/licenses/by/4.0/ This content is distributed under the terms of the Creative Commons Attribution 4.0 International license.

10.1128/mbio.00764-22.4 FIG S4 Atypical HK enriched clusters in four *Campylobacterota* species with abundant TCS genes. Each genome is linearized and depicted as a scale line, and signal transduction genes are represented as colored lines based on their starting location. The HHK and HRR enriched regions are illustrated at the bottom. Download FIG S4, PDF file, 0.5 MB. Copyright © 2022 Mo et al. 2022 Mo et al. https://creativecommons.org/licenses/by/4.0/ This content is distributed under the terms of the Creative Commons Attribution 4.0 International license.

Although the abundance of TCSs can be quantified, their evolutionary modes cannot be precisely measured. We found that fusion of duplication and HGT pieces produces many chimeric HKs and RRs with non-DNA-binding domain outputs. Meanwhile, it is challenging to distinguish between vertical inheritance after an earlier HGT event with many gene losses and the recent HGTs among closely related species, especially with the avalanche of genomic data nowadays. In spite of these difficulties in quantification of gene duplication and HGT ratios, it is generally accepted that both of them provide the raw materials to expand TCS pathways. During bacterial niche expansion, massive gene duplications and HGTs might easily result in new domain organizations of signaling proteins that are highly modular. Thus, more atypical HKs, including HHKs and HRRs, and more atypical RRs with non-DNA-binding domain outputs emerge while the TCS size increases. These lead to the Matthew effect in signaling pathways, “the rich get richer and the poor get poorer.” Our results suggest that REC domains promote connectivity as well as genetic innovation to increase the complexity of TCS.

### C-di-GMP is made for connection.

**FIG 3:**
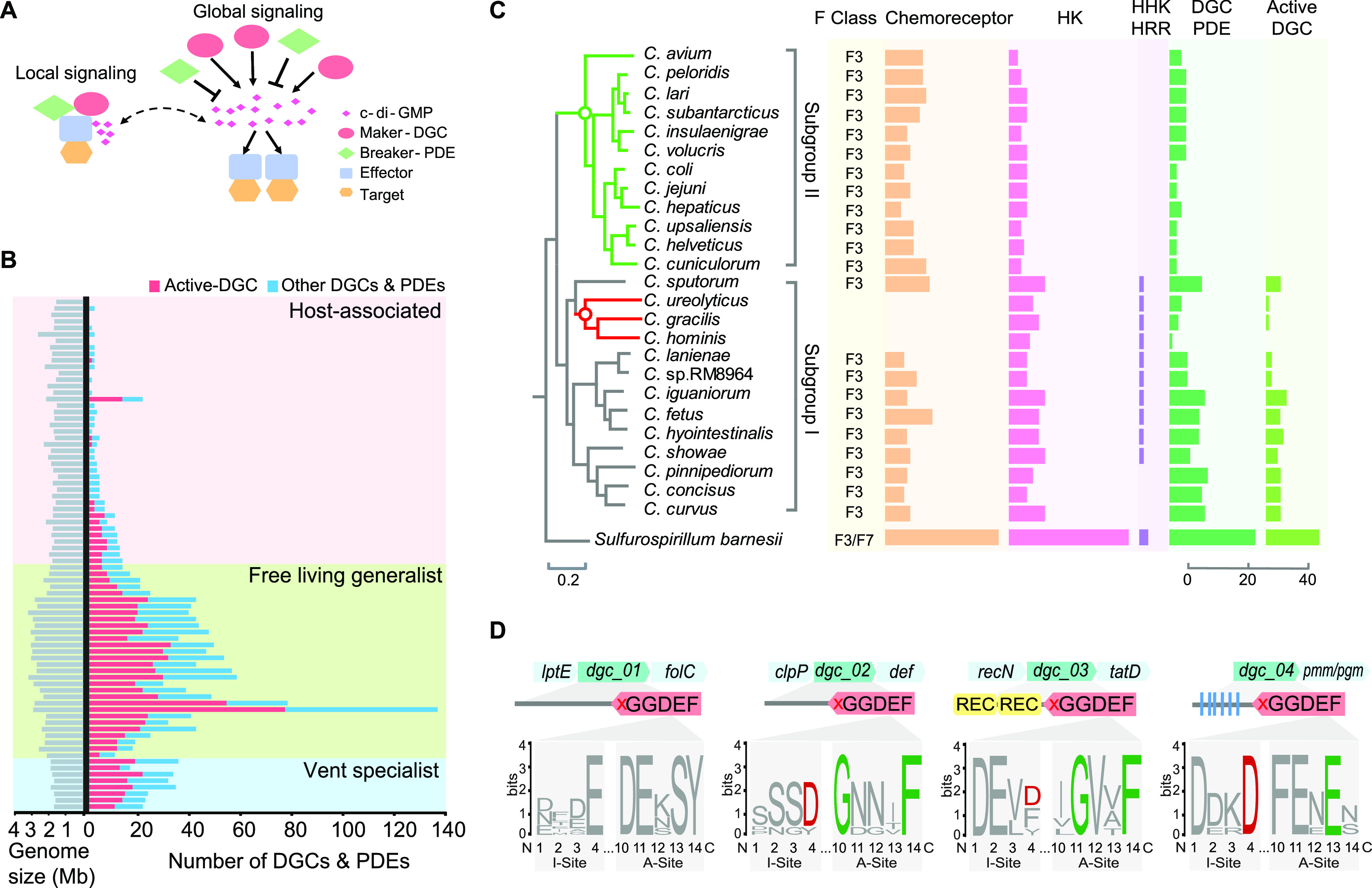
C-di-GMP-mediated system in *Campylobacterota*. (A) Local and global c-di-GMP signaling system. (B) The abundance of DGCs and PDEs in *Campylobacterota* species. The category and number of DGCs and PDEs in each genome are in Table S4 at https://data.mendeley.com/datasets/wxwcjzm9wv/1. (C) The number of signaling proteins in the Campylobacter genus. The phylogenetic tree is taken from Fig. S1. In the tree, green circle represents the branch point of subgroup II when the c-di-GMP signaling system is lost; red circle represents the branch point of a lineage losing flagellar genes. (D) Four inactive DGCs in species of subgroup II in panel C. The flanking genes of these 4 DGCs are indicated above, and their domain organization, degenerate enzymatic (A-site), and c-di-GMP binding (I-site) motifs are shown below. Active I-site generally features RxxD, and the active A-site is characterized by GGD/EEF, so the GGDEF domains of the 4 DGCs here are marked by a red cross, implying enzymatic inactivity.

C-di-GMP is the most ubiquitous secondary messenger in bacteria. It is produced from two GTP molecules by diguanylate cyclases (DGCs) with GGDEF domain broken down by phospodiesterases (PDEs) that contain either EAL or HD-GYP domain (40). The core of the c-di-GMP signaling system is composed of enzymes that make and break it and diverse effectors that it can bind to regulate biofilm, motility, cell cycle, virulence, etc. (41). The complexity of this system has long been realized but not yet understood, owing to the inherent “many to many” links connected by this single molecule (Fig. 3A) (42). Global c-di-GMP regulation mode consists of input signal integration and output divergence based on the diffusible nature of this molecule and the fact of “more enzymes, less effectors” being identified in many species. In contrast, local c-di-GMP signaling pathways employ physical interaction or complex formation of specific DGC, PDE, effector, and target to ensure specificity. Moreover, both local and global regulation can form dynamic links with spatiotemporal control (43, 44).

Due to the lack of knowledge of all the effectors, we assessed the complexity level of c-di-GMP-mediated system by the quantification of its turnover enzymes. DGCs and PDEs can be readily identified by their dedicated domains (GGDEF, EAL, or HD-GYP domains). Our domain analysis of all DGCs and PDEs revealed that 98% are multimodule, with N-terminal sensory domains in common with chemoreceptors and HKs or with REC domains mostly having cognate HKs in close proximity (Fig. S5). Thus, the number of c-di-GMP enzymes can represent the sensing ability of the species. In addition, for species with multiple c-di-GMP enzymes, their sensory domains or connected sensory proteins (such as HKs) are different, thus no functional redundancy exists for these enzymes (Fig. S5). However, not all identified DGCs and PDEs are active, and enzymatically inactive GGDEF or EAL domains often serve as c-di-GMP binding effectors (45). If no active DGC is encoded by the genome, no c-di-GMP can be produced to mediate signal transduction, even in the presence of degenerate domains. Here, we separate the proteins with enzymatically active GGDEF domain, called active DGCs, from all the other proteins containing EAL, HD-GYP, or degenerate GGDEF domains, called the rest of the DGCs and PDEs (see Table S4 at https://data.mendeley.com/datasets/wxwcjzm9wv/14).

10.1128/mbio.00764-22.5 FIG S5 Domain architectures of all DGCs and PDEs in representative species of each genus within the *Campylobacterota* phylum. These proteins are categorized as transmembrane and cytoplasmic proteins. The red asterisk represents a DGC or PDE gene with a REC domain, also in close proximity of a HK gene. The red cross in the GGDEF domain implies an enzymatically inactive DGC. In the box of Sulfurimonas gotlandica GD1, two pairs of DGCs are highlighted in red, indicating recent gene duplication of two genes. Download FIG S5, PDF file, 2.7 MB. Copyright © 2022 Mo et al. 2022 Mo et al. https://creativecommons.org/licenses/by/4.0/ This content is distributed under the terms of the Creative Commons Attribution 4.0 International license.

The total number of DGCs and PDEs within *Campylobacterota* species varies greatly, ranging from 0 to 137, indicating that bacterial genomes gain and lose c-di-GMP enzymes with ease (Fig. 3B). Vent specialists generally have >20 c-di-GMP turnover enzymes, more than their average number of HKs or chemoreceptors, implying that c-di-GMP signaling pathways play an important role in ancient lineages. Free-living generalists gain more enzymes, with upsurge in two species but no big increase of their genome size (Fig. 3B; see also Table S4 at https://data.mendeley.com/datasets/wxwcjzm9wv/1). This extreme proliferation of DGCs and PDEs is accompanied by F3/F7/F9 chemosensory classes and a larger number of TCS genes in these two species (Fig. 1A and 2B), indicative of many gene gain events of signaling systems *en masse* during niche adaptation. The sheer abundance of DGCs and PDEs drops abruptly in host-associated species (Fig. 3B). No active DGC could be found in the *Helicobacter* genus except for one species and more than half of the species of the Campylobacter genus, meaning that c-di-GMP signaling system had disappeared from these host-associated species (see Table S4 at https://data.mendeley.com/datasets/wxwcjzm9wv/1). Overall, the number of c-di-GMP enzymes in most *Campylobacterota* species correlates with their ecotype range (Fig. 3B).

The ubiquity of c-di-GMP enzymes within *Campylobacterota* suggests that the c-di-GMP signaling system evolved in the ancestor of this phylum and greatly expanded in free-living generalists during niche expansion. The upsurge of c-di-GMP enzymes to 137 in *S*. *gotlandica* GD1 compared to 30 to ~40 in other species of the same genus raises an interesting question. How does a bacterium quickly gain so many gene copies of c-di-GMP enzymes? Sequence analyses of all 137 c-di-GMP enzymes revealed only two pairs of proteins with identical domain organization and high sequence similarity, suggestive of recent gene duplications (Fig. S5). Other enzymes with highly similar GGDEF domains have different types of sensory domains or show diverse combinations of sensory domains, GGDEF and EAL domains, unlikely to be produced by a single-gene duplication event (Fig. S5). Thus, the upsurge of c-di-GMP enzymes in bacterial species might be achieved by mixed evolutionary modes: both HGTs and gene duplications provide the genetic material, and gene fusion/fission and fast sequence divergence contribute to make new genes.

Another intriguing question is the absence of c-di-GMP signaling system in host-associated pathogens such as Helicobacter pylori and Campylobacter jejuni, since c-di-GMP plays an important role in regulation of virulence in many other pathogens. To approach the determinants of c-di-GMP signaling pathways, we examined the gradual change of all signaling proteins for the Campylobacter genus based on our eco-evo framework. All Campylobacter species have greatly reduced numbers of components for all signal transduction systems compared to its close relative, *Sulfurospirillum* genus, suggesting that substantial gene loss of signaling proteins happened for the last common ancestor of the Campylobacter genus, likely due to the association with animal hosts (Fig. 3C). Within this genus, two distinctive clusters can be defined: subgroup I with relatively more signaling proteins, such as TCSs and c-di-GMP enzymes, and subgroup II with fewer signaling proteins and no canonical active DGCs. To verify the absence of active DGCs in subgroup II, we further examined 443 strains of Campylobacter species with completely sequenced genomes (see Table S5 at https://data.mendeley.com/datasets/wxwcjzm9wv/1). For subgroup II, all the genomes still encode 1 to ~4 proteins with degenerate GGDEF domains and do not possess an active GGDEF domain or EAL/HD-GYP domains (see Table S5 at https://data.mendeley.com/datasets/wxwcjzm9wv/1). Importantly, these degenerate GGDEF domains within subgroup II also have degenerate inhibitory sites for c-di-GMP binding, indicating that they are unlikely to serve as c-di-GMP effectors either (Fig. 3D). Furthermore, the species of subgroup II only retain 4 to ~6 TCS pairs but no HHK or HRR, one F3 chemosensory class, only one STYK found in one species, and no cAMP-mediated pathways or ECFs (Fig. 3C). Except for the single chemosensory class for flagellar motility, other signal transduction systems in these species are basically OCS proteins and very few TCS pairs, lacking components and links compared to the subgroup I with active DGCs. Hence, it is possible that the presence of c-di-GMP signaling system requires a minimal signal transduction network size or connectivity to build more links into it.

### The evolution of other signaling systems in *Campylobacterota*.

**FIG 4:**
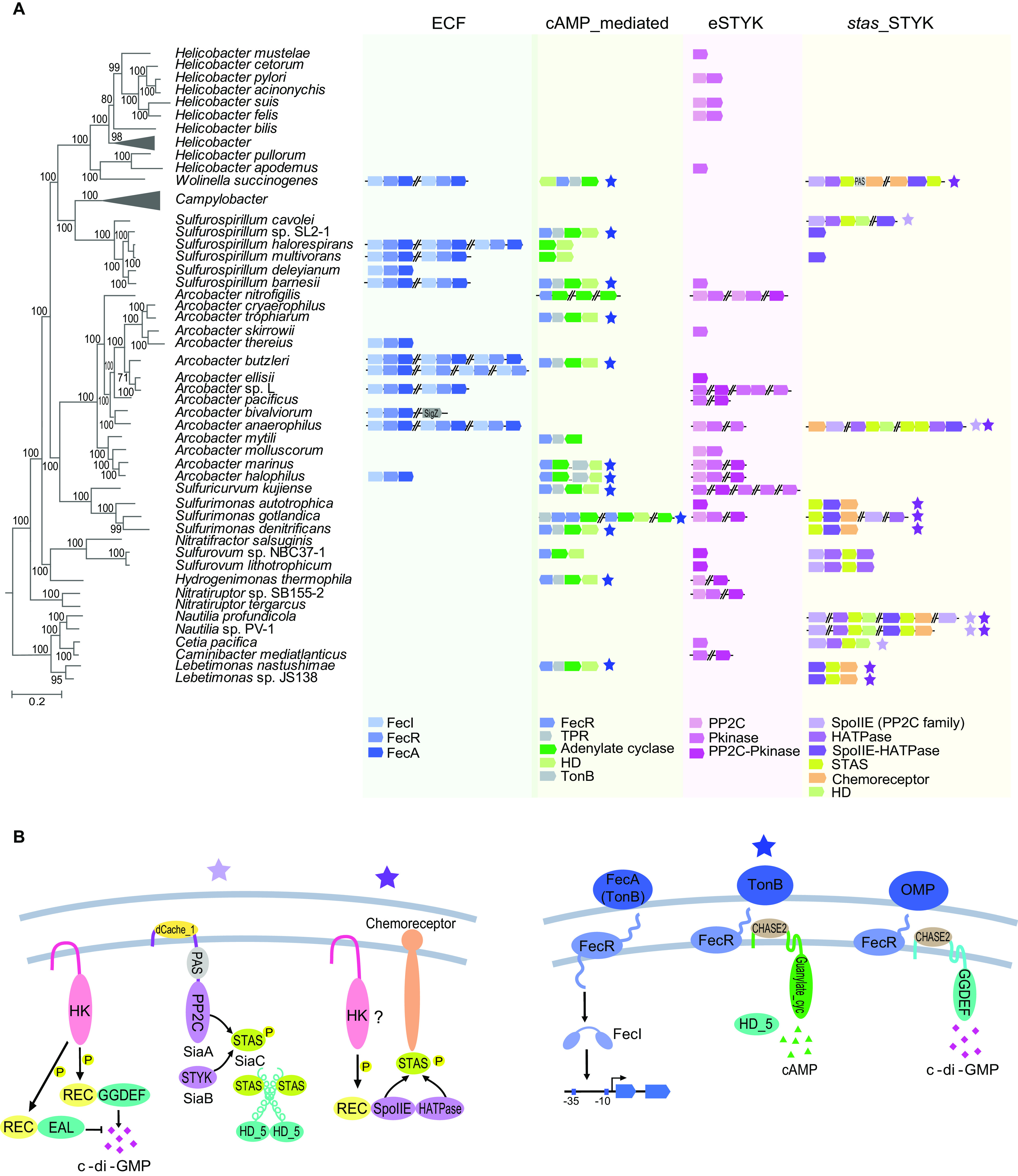
ECF, cAMP, and STYK systems in *Campylobacterota*. (A) Phylogenetic profile of ECF, cAMP, and STYK systems in *Campylobacterota.* Genes or gene clusters constituting the ECF, cAMP, or STYK systems are shown for species that contain them. The Campylobacter genus and partial *Helicobacter* species that do not have ECF, cAMP, or STYK signaling systems are collapsed. SpoIIE protein or SpoIIE domain that dephosphorylates STAS protein belongs to PP2C family. (B) Potential pathways with combination of two or more systems. (Left) Examples of potential pathways found in Nautilia profundicola AmH. (Right) Classical ECF pathway and ECF components potentially interacting with cAMP or c-di-GMP enzymes in Arcobacter halophilus CCUG 53805. Stars with different colors are also labeled in panel A, indicating the presence of the potential pathways in the species.

ECFs are typically regulated by their cognate anti-σ factors, which can sense environmental stimuli and guide the RNA polymerase to initiate expression of genes related to stress responses (46). Recent census studies of ECF proteins greatly expanded their known diversity, and they were reclassified into 157 groups (18). However, only 2 ECF groups were identified in several *Campylobacterota* species. One ECF group, ECF238 (also called SigZ), is found in just one *Arcobacter* species, acquired through HGT from deltaproteobacteria; the other group, ECF242, is present in 3 genera, including *Arcobacter*, *Sulfurospirillum*, and *Wolinella* (Fig. 4A; also see Table S6 at https://data.mendeley.com/datasets/wxwcjzm9wv/1). The presence of ECFs in these more recently evolved genera suggests that the ECF system was introduced into *Campylobacterota* during niche expansion by HGT. ECF242 is composed of a σ factor, FecI, an anti-σ factor, FecR, and a TonB-dependent receptor, FecA (18). Only half of the species of the above-described 3 genera have ECF242 gene cluster, and most of them contain 2 or more copies (Fig. 4A). Phylogenetic analyses of FecI and FecR protein do not show the same topology as the species tree, indicating that HGT events happened multiple times among these 3 genera alongside gene duplications (Fig. S6). Nevertheless, FecI and FecR protein trees share the same clustering pattern, supporting the coevolution of an ECF σ factor and its anti-σ factor.

10.1128/mbio.00764-22.6 FIG S6 Phylogenetic tree of FecI (a) and FecR (b) proteins in the *Campylobacterota* phylum. Download FIG S6, PDF file, 0.4 MB. Copyright © 2022 Mo et al. 2022 Mo et al. https://creativecommons.org/licenses/by/4.0/ This content is distributed under the terms of the Creative Commons Attribution 4.0 International license.

cAMP is produced by adenylate cyclase and binds cAMP receptor protein (CRP) or a few other proteins to control diverse cellular functions (47). Only 21% of *Campylobacterota* species encode adenylate cyclase, but 86% of species have one or more CRP-like proteins, indicating that CRP homologs in the species without adenylate cyclase are not likely to be regulated by cAMP (see Table S6 at https://data.mendeley.com/datasets/wxwcjzm9wv/1). Notably, all adenylate cyclases identified in *Campylobacterota* species have the same sensory domain (CHASE2 domain), and most of them are located in a cluster containing a phosphodiesterase that can hydrolyze cAMP, an FecR domain-containing outer membrane protein, and a TPR domain-containing protein (Fig. 4A). The clustering of these four genes in multiple *Campylobacterota* species suggests that they participate in the same signal transduction pathway. Phylogenetic analysis of the conserved adenylate cyclase did not yield the same branch pattern as the species tree (Fig. S7), indicative of HGT events of this gene cluster among free-living *Campylobacterota* species as well as other bacterial phyla.

10.1128/mbio.00764-22.7 FIG S7 Phylogenetic tree of adenylate cyclase in the *Campylobacterota* phylum. Pink, 3 adenylate cyclase homologs in Sulfurimonas gotlandica GD1; blue, 3 adenylate cyclase homologs in Arcobacter nitrofigilis DSM 7299. Download FIG S7, PDF file, 0.3 MB. Copyright © 2022 Mo et al. 2022 Mo et al. https://creativecommons.org/licenses/by/4.0/ This content is distributed under the terms of the Creative Commons Attribution 4.0 International license.

STYKs that can phosphorylate serine, threonine, or tyrosine residues in target proteins are of two kinds in bacteria, and they are evolutionarily unrelated (48). One kind is eukaryotic-type protein kinases (eSTYKs), characterized by the presence of Pfam’s Pkinase domain (PF00069); the other kind is members of histidine kinase-like ATPase family, but they phosphorylate serine/threonine residues in specific proteins with a single STAS domain (also called anti-sigma factor antagonists). Here, we name the 2nd kind of STYKs with HATPase domain but no HisKA domain (thus, not HK of TCS) as *stas*_STYKs. Both eSTYKs and *stas*_STYKs share the same partner phosphatase, PP2C family phosphatase, to reverse the phosphorylation reaction. One-third of *Campylobacterota* species have eSTYKs, either an eSTYK and a cognate PP2C phosphatase or a bifunctional kinase/phosphatase fusing PP2C and Pkinase domains together (Fig. 4A; see also Table S6 at https://data.mendeley.com/datasets/wxwcjzm9wv/1). Only one-fifth of the *Campylobacterota* species have *stas*_STYKs genes, mostly having target *stas* and cognate PP2C phosphatase genes in the same neighborhood in the genome (Fig. 4; see also Table S6 at https://data.mendeley.com/datasets/wxwcjzm9wv/1). Except for their clustering pattern with cognate phosphatases, the species distribution of both eSTYKs and *stas*_STYKs in *Campylobacterota* is sporadic, apparently resulting from multiple HGT events. For example, the eSTYKs and their phosphatase genes in 7 *Arcobacter* species reside in the middle of type VI secretion system loci, which are prone to HGT (see Table S6 at https://data.mendeley.com/datasets/wxwcjzm9wv/1).

Taken together, in contrast to the prevalence of chemosensory, TCS, and c-di-GMP systems in *Campylobacterota* species, ECF, cAMP, and STYK systems are mostly present in free-living generalists with sporadic distribution. Our phylogenetic analyses suggest that the last common ancestor of *Campylobacterota* already evolved F3 chemosensory class, TCS, and c-di-GMP systems; thus, vertical inheritance, gene duplication, and HGT can greatly expand these ancestral systems and result in increased complexity. However, ECF, cAMP, and STYK systems apparently were acquired multiple times by HGT and were mostly lost in host-associated Campylobacter and *Helicobacter*. Aside from the 6 signaling systems described above, the *Campylobacterota* phylum does not have c-di-AMP and QS systems. It should be mentioned that QS differs from all other signal transduction systems in terms of generally sensing self-derived or other microbe-derived rather than environmental signals. Although none of the known QS pathways has been identified in any *Campylobacterota* species, our domain analyses of known QS components in model organisms from other phyla indicate that QS receptors belong to OCS, TCS, c-di-GMP enzymes, or chemoreceptors (Fig. S8). Thus, we conclude that QS adopts existing modules to merge into the signaling network without *de novo* innovation in transmitter type or mechanism.

10.1128/mbio.00764-22.8 FIG S8 Domain analyses of proteins in QS pathways. QS pathways are sorted by QS molecules; reported synthetases and receptors are listed. The ligand binding domains for QS molecules in the receptors are highlighted in orange. All proteins listed in the table are from non-*Campylobacterota* species that have been experimentally studied for the specific QS pathway. Download FIG S8, PDF file, 0.3 MB. Copyright © 2022 Mo et al. 2022 Mo et al. https://creativecommons.org/licenses/by/4.0/ This content is distributed under the terms of the Creative Commons Attribution 4.0 International license.

### Complexity beyond each signaling system.

Theoretically, the modularity of signaling proteins enables the merging of different mechanisms into one pathway, and bacteria had already used this strategy to assemble multistep information processing during evolution. As described earlier, TCSs are frequently merged with other systems through the fusion of REC domain with noncanonical output domains. In addition, one of the chemosensory classes in Pseudomonas aeruginosa is reported to regulate c-di-GMP concentration because its response regulator is fused with GGDEF domain (49). Our analyses also discovered potential pathways combined with two or more systems, based on neighborhood gene cluster, known protein-protein or domain-domain interactions, and signaling flow logic from sensors to transmitters (Fig. 4B). For example, based on their cooccurrence in multiple *Campylobacterota* species, a PP2C phosphatase with periplasmic sensory domain and a *stas*_STYK probably regulate a c-di-GMP turnover enzyme by the phosphorylation and dephosphorylation of a STAS protein. Similarly, a bifunctional PP2C-STYK protein with N-terminal REC domain that is likely regulated by an unknown HK protein itself might affect a chemoreceptor conformation by its target STAS protein (Fig. 4A and B; see also Table S6 at https://data.mendeley.com/datasets/wxwcjzm9wv/1). These potential pathways in *Campylobacterota* species lack experimental verification so far, but the merge of STYK-PP2C and c-di-GMP systems has been recently reported as the SiaABCD pathway in P. aeruginosa (50, 51). This pathway is an example of a c-di-GMP enzyme (SiaD) regulated by PP2C (SiaA), *stas*_STYK (SiaB), and STAS (SiaC) proteins. Another recent study proposed a working model for ECF modules for inhibition of transmembrane cAMP enzyme activity (52). Our analyses revealed clustering of genes encoding ECF sensors, partial ECF transmitters, and transmembrane cAMP or c-di-GMP enzymes in multiple *Campylobacterota* species, suggesting their functional relationship (Fig. 4A and B; see also Table S6 at https://data.mendeley.com/datasets/wxwcjzm9wv/1). The common theme of these potential pathways is to convert extracellular signals to intracellular secondary messengers or protein phosphorylation-based transmission processes, perhaps allowing for multiple input signal integration and output divergence (Fig. 4B). Future experimental validations are needed for these pathways with combined systems, which will provide insights for the development of sophisticated genetic circuits in synthetic biology.

## DISCUSSION

Here, we defined the complexity of each system differently based on their signal transduction mechanism, which determines how basic interactions among components are formed and more interactions can be propagated. Thus, we cannot simply group signal transduction systems such as OCS, TCS, and others but rather recognize their distinctive mechanisms and then disentangle the network evolution. In the eco-evo context of the *Campylobacterota* phylum, we compared the dynamic changes of all signaling systems and revealed that bacterial signaling capacity and complexity rise and drop together.

**FIG 5:**
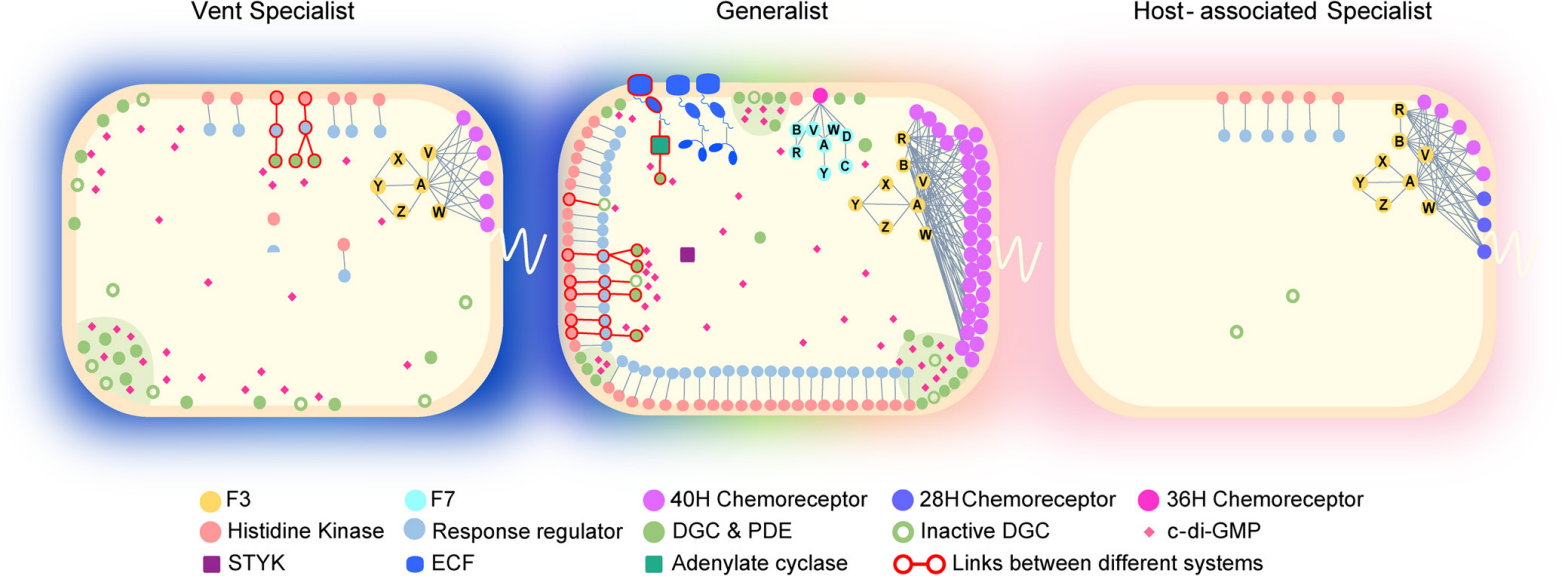
Signaling networks in representative species from diverse ecological niches. Vent specialist, Lebetimonas natsushimae HS1857; generalist, Sulfurospirillum barnesii SES-3; host-associated specialist, Campylobacter jejuni 81-176. The shading color around the cell depicts the ecotype range of the representative species: blue, deep-sea hydrothermal vent; pink, host associated. Iridescence indicates various environments. Transmembrane DGCs and PDEs are arbitrarily clustered within cell to represent local c-di-GMP signaling.

Niche expansion of the species leads to a larger genetic pool from the microbial community, which facilitates HGT; meanwhile, stresses encountered along with environmental changes may cause mistakes during DNA replication followed by repair, resulting in gene duplication, loss, fusion, and fission. These genetic events not only propagate existing protein interactions but also create opportunities for innovation of new genes/pathways and combination of different mechanisms in one signaling pathway. Thus, free-living generalists with large signaling networks tend to employ multistep or branched pathways to achieve tunable information processes (Fig. 5). On the contrary, when species become specialized in a restricted habitat, a large sensory repertoire is no longer required but can be energy costly. Thus, signaling networks shrink together with their regulated targets or pathways, leading to a streamlined genome for specialist lifestyle. Moreover, host-associated specialists generally retain simple OCSs and classical TCSs but lose complex pathways with multiple components and many links (Fig. 5). The gradual disappearance of c-di-GMP system and other systems in the Campylobacter genus provides such an example. However, if the signal outputs are retained in specialists, their coevolved signaling systems are still needed. For example, both H. pylori and C. jejuni, with very streamlined genomes and minimal signaling network size, still retain multicomponent chemosensory systems to control their flagellar motility (Fig. 5). Thus, host-associated specialists tend to keep their signal transduction simple and straightforward in a small network, but they often show biased preference for signaling systems determined by coevolution with the outputs.

All links in the signaling network are based on protein-protein or protein-ligand interactions. To ensure signaling specificity, coevolution of interactive partners is required to avoid nonspecific cross talk, which is best exemplified by classical TCSs (53, 54). Actually, coevolution exists at all protein-protein interactions in all of the signal transduction systems, including chemosensory classes, c-di-GMP local signaling complexes, ECF σ factors and anti-σ factors, *stas*_STYKs, and STASs. Coevolution constraints normally apply to the interaction interfaces of components within one pathway. If many interactions are involved in signal transmission, many proteins need to coevolve, and as a result, their evolutionary flexibility will decrease and vice versa. Hence, multicomponent chemosensory system generally adds new pathways by acquiring entire chemosensory classes through HGT, but simpler signaling systems with two or fewer components can gain more transmitters by both gene duplication/divergence and HGT. However, secondary messenger such as c-di-GMP can break the coevolution constraint if the link between an enzyme and an effector depends solely on this same molecule with no direct protein-protein interactions involved. Due to this flexibility, a c-di-GMP enzyme gained by gene duplication or HGT can directly generate links with existing effectors. Likewise, if a c-di-GMP enzyme loses its function, its effector(s) can be easily rewired by other existing enzymes to complement its function under selection, as demonstrated by laboratory evolution experiments (55, 56). This might explain why bacterial species with c-di-GMP-mediated system gain and lose c-di-GMP enzymes very dynamically compared to other signaling systems.

Finally, it is extremely difficult to delineate the signaling pathways for a bacterial species with dozens of signaling homologs at its disposal. Our protein domain analyses suggest that the activities of transmitter domains are directly or indirectly regulated by different sensory domains responding to diverse stimuli. Hence, no functional redundancies really exist for signaling homologs in one bacterial cell with a complex network.

## MATERIALS AND METHODS

### Data sources.

The statistics of completely sequenced genomes of various bacterial phyla were collected in NCBI RefSeq database, and the taxonomic ranks for some new phyla were updated according to GTDB database (see Table S1 at https://data.mendeley.com/datasets/wxwcjzm9wv/1) (24, 25). For *Campylobacterota* species with multiple completely sequenced strains, only one strain was selected to represent the species. To cover the most species diversity and also ensure genome quality, 74 complete genomes and 8 incomplete but close to complete genomes were included for analyses (see Table S2 at https://data.mendeley.com/datasets/wxwcjzm9wv/1). All 82 genome sequences were downloaded from the NCBI genome database (https://www.ncbi.nlm.nih.gov/genome/microbes/). The ecological and physiological information of all strains were extracted from references listed in Table S2 at https://data.mendeley.com/datasets/wxwcjzm9wv/1. For analyses of c-di-GMP enzymes in the Campylobacter and *Helicobacter* genera, all completely sequenced genomes from both genera were downloaded from the same NCBI genome database. The numbers of complete genomes for each Campylobacter or *Helicobacter* species are summarized in Table S5 at https://data.mendeley.com/datasets/wxwcjzm9wv/1).

### Bioinformatics software.

Phylogenetic trees were constructed either by FastTree 2.0 or Mega X programs as indicated below (57, 58). All homologous protein searches were conducted by blast-2.12.0 (59); multiple-sequence alignments were performed by MAFFT program using the e-ins-i algorithm (60); domain architectures of all proteins were identified by SMART and HHpred (61, 62). TreeCollapse was used to produce the final tree figure in Fig. 1D with a large data set (63). Sequence logos for multiple-sequence alignments shown in Fig. 3D were generated by Weblogo (64).

### Phylogenomic tree construction for *Campylobacterota*.

Eighty-two genomes from *Campylobacterota* and 6 genomes from closely related *Desulfurellales* class as the outgroup were retrieved to reconstruct the species tree for the *Campylobacterota* phylum. Phylogenetic inference was performed using the UBCG pipeline (65). Basically, 92 single-copy orthologous genes were identified from these genomes by Prodigal and HMMER with default values (66, 67). Orthologous protein sequences were aligned by MAFFT, and the alignment positions with gap characters from more than 50% of sequences were excluded by Gblocks (68). A maximum likelihood tree based on concatenated protein sequence alignments was constructed in FastTree using the JTT + CAT model, and bootstrap analysis was carried out using 1,000 replications.

### Analyses of chemosensory system.

All protein components of the chemosensory system (CheA, CheW, CheV, CheB, CheR, CheC, CheD, CheX, CheY, CheZ, and chemoreceptors) were identified by blast-2.12.0 against 82 *Campylobacterota* genomes. The blast hits with E values of less than e−5 were assigned as potential homologs, and all the candidates were reexamined by searching at MiST, SMART, and HHpred databases manually (19). The chemosensory genes were mapped on the linearized genomes and visualized by RIdeogram (69). The chemosensory F class was assigned based on reference 30. The H classes for all chemoreceptors were manually assigned based on reference 70 and also verified using MiST database (19).

A maximum-likelihood phylogenetic tree was constructed based on concatenated sequence alignments of CheV, CheA, and CheW of F3 class from *Campylobacterota* genomes. The tree was constructed in MEGA X using WAG model with 100 bootstrap replications. Another phylogenetic tree based on 2,136 CheA homologs of different F classes from various bacterial phyla (1,354 species) was constructed by FastTree using JTT + CAT model.

### Analyses of TCS.

Both HisKA (Pfam no. PF00512) and HATPase (PF02518) domains were used as query sequences for searching HKs and REC (PF00072) domain as the query for RRs in each genome by blast-2.12.0. In addition, if a REC domain is found at the C terminus after or N terminus before both HisKA and HATPase domains in a protein sequence, it was considered HHK or HRR, respectively. A solo HK or HHK gene is more than four genes away from an RR gene. The classification of TCS proteins in each genome is presented in Table S3 at https://data.mendeley.com/datasets/wxwcjzm9wv/1. The gene neighborhood of TCS genes was manually checked in selected species with abundant HHK and HRR genes. All signal transduction genes were plotted on the linearized genomes of these species by RIdeogram. The domain architectures of all HKs and RRs in representative species of each genus are shown in Fig. S4 in the supplemental material.

### Analyses of DGCs and PDEs.

We identified all DGCs and PDEs by BLAST searching GGDEF (PF00990), EAL (PF00563), and HD-GYP (PF13487) domains against all 82 *Campylobacterota* genomes according to the characteristics of DGCs and PDEs summarized in reference 40. Protein domain architecture was analyzed by SMART. DGCs with active GGDEF domain should have GGDEF/GGEEF/SGDEF/AGDEF motif at the A-site; otherwise they were considered degenerate DGCs. We classified all the DGCs and PDEs into 6 categories in each species: total DGCs; DGCs with partner domain (potential sensory domain or domain mediating interaction with another protein); PDEs with partner domain; bifunctional GGDEF-EAL/HD-GYP domain-containing proteins; single-domain DGCs and PDEs; and DGCs with active GGDEF domain (see Table S4 at https://data.mendeley.com/datasets/wxwcjzm9wv/1). The domain architectures of DGCs and PDEs in representative species of each genus are shown in Fig. S5.

### Analyses of ECF, cAMP, and STYK systems.

ECF σ factors were identified using sigma70_r2 (PF04542) and sigma70_r4_2 (PF08281) domains as the query for BLAST search, and only BLAST hits with both and only these two domains were counted as ECF σ factors according to reference 18. The gene neighborhood was manually analyzed to determine the ECF groups according to reference 18. Phylogenetic trees of FecI and FecR proteins in *Campylobacterota* were constructed by Mega X by following the methods described above.

Both Adenylate_cycl (PF01295) and Guanylate_cyc (PF00211) domains were used as the query for BLAST search of adenylate cyclase in each genome. Phylogenetic trees of adenylate cyclases in *Campylobacterota* were constructed by following the method described above, and the gene neighborhood was manually checked.

For identification of eSTYKs systems, the Pkinase (PF00069) and PP2C (PF00481) domains were used as query to perform BLAST search. For *stas*_STYKs systems, the SpoIIE (PF07228) and HATPase (PF13581) domains were used as query.

All protein identifiers of these 3 systems are summarized in Table S6 at https://data.mendeley.com/datasets/wxwcjzm9wv/1.

### Analyses of QS pathways.

The enzymes and receptors of six well-studied QS pathways in representative strains from previous studies are summarized in Fig. S8. The domain structures of both synthetases and receptors were identified using SMART. The protein sequences of synthetases and the potential ligand binding domains of the receptors were used as probes to do BLAST searches against *Campylobacterota* genomes. None of the genomes returned hits for synthetase and corresponding receptor of each QS pathway. Thus, we conclude that none of the QS pathways is present in analyzed genomes of the *Campylobacterota* phylum.
