# Supplementary material for: Evolutionary Principles of Bacterial Signaling Capacity and Complexity
Source: mBio. 2022 May 10;13(3):e00764-22. doi: 10.1128/mbio.00764-22 (PMC9239204; doi:10.1128/mbio.00764-22)
Supplement: FIG S1 [file mbio.00764-22-sf001.pdf]

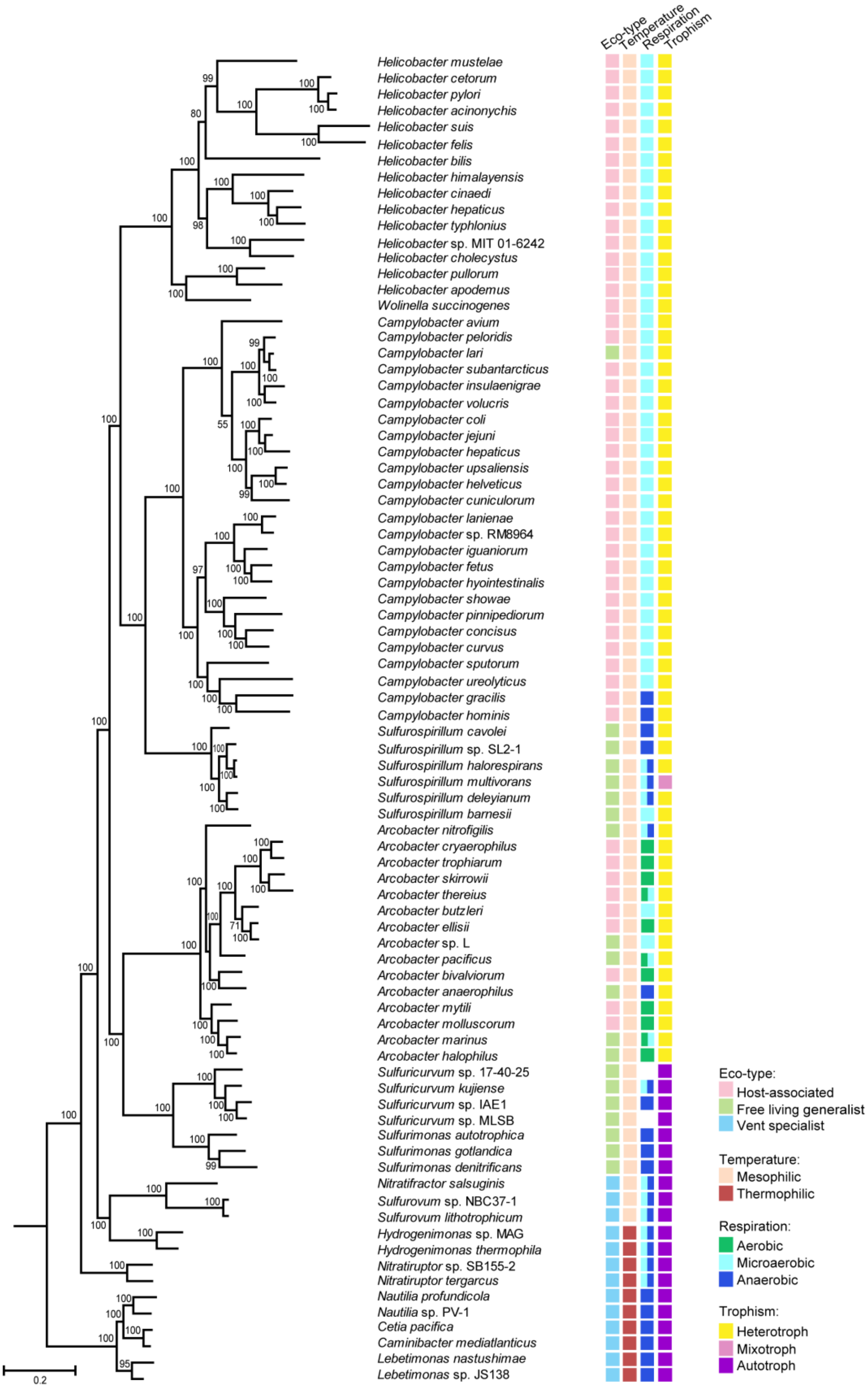

Fig. S1. Phylogenetic tree and species ecophysiological characteristics of the *Campylobacterota* phylum. Detail information of all species in Table S2 (see Table S2 at https://data.mendeley.com/datasets/wxwcjzm9wv/1).
