# Supplementary material for: Evolutionary Principles of Bacterial Signaling Capacity and Complexity
Source: mBio. 2022 May 10;13(3):e00764-22. doi: 10.1128/mbio.00764-22 (PMC9239204; doi:10.1128/mbio.00764-22)
Supplement: FIG S2 [file mbio.00764-22-sf002.pdf]

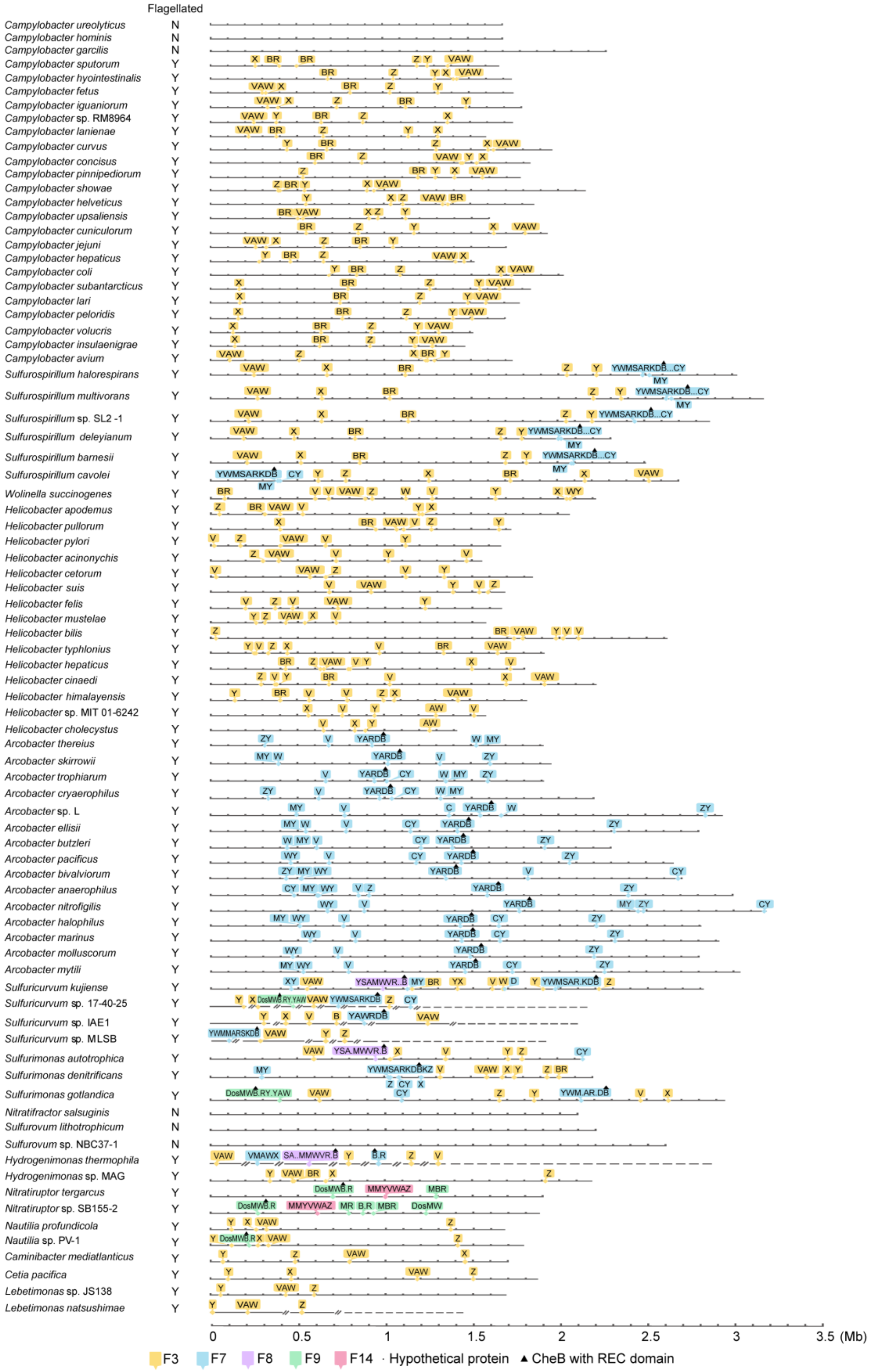

Fig. S2. Chemosensory classes of *Campylobacterota* species are illustrated in linearized genomes. The color codes of chemosensory classes are noted in the bottom panel; C, cheC; X, cheX; Z, cheZ and other gene abbreviations are the same as Fig. 1B.
