# Supplementary material for: Evolutionary Principles of Bacterial Signaling Capacity and Complexity
Source: mBio. 2022 May 10;13(3):e00764-22. doi: 10.1128/mbio.00764-22 (PMC9239204; doi:10.1128/mbio.00764-22)
Supplement: FIG S3 [file mbio.00764-22-sf003.pdf]

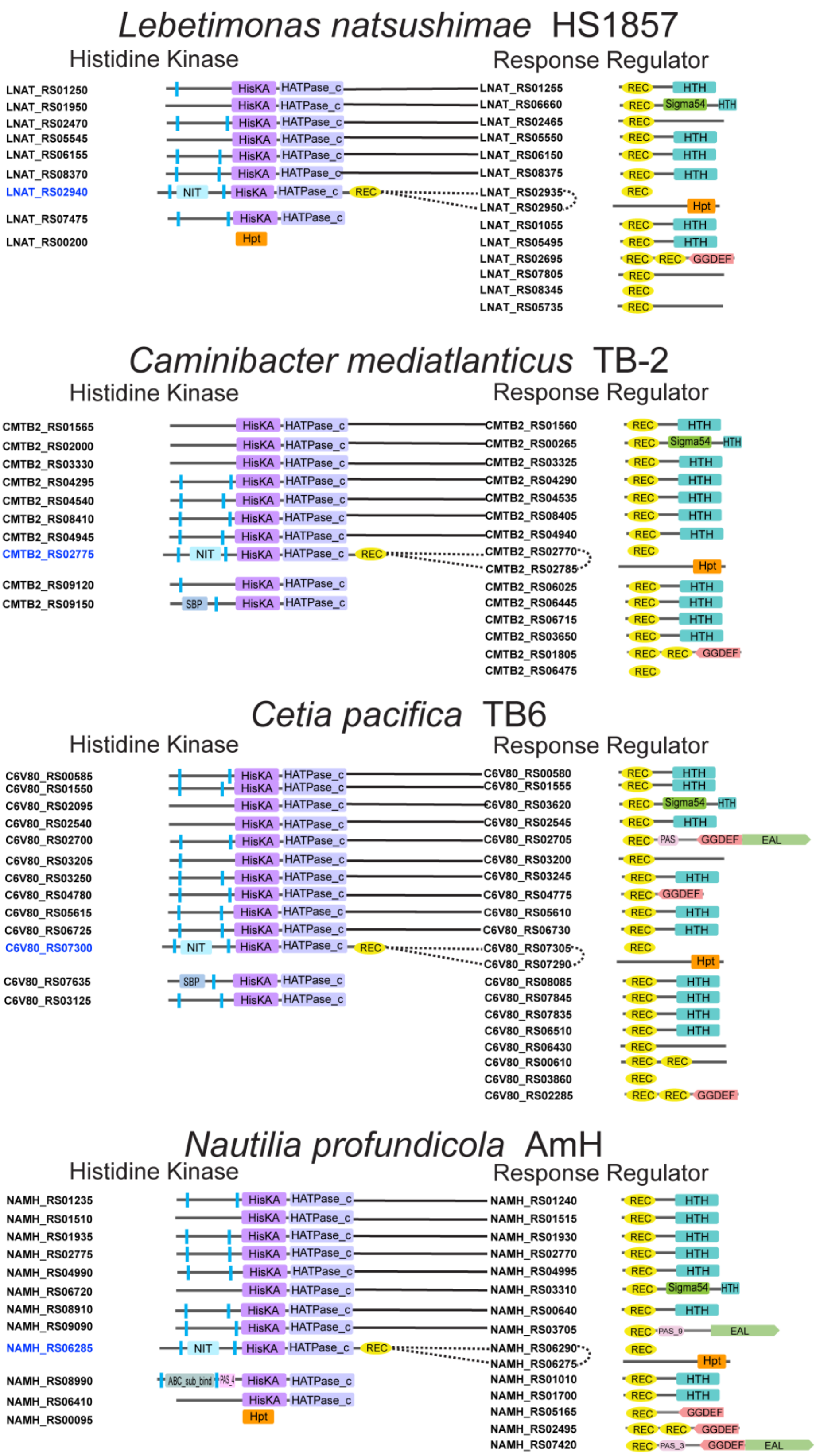
Lebetimonas natsushimae HS1857
Histidine Kinase
Response Regulator
LNAT_RS01250
LNAT_RS01950
LNAT_RS02470
LNAT_RS05545
LNAT_RS06155
LNAT_RS08370
LNAT_RS02940
LNAT_RS07475
LNAT_RS00200
LNAT_RS01255
LNAT_RS06660
LNAT_RS02465
LNAT_RS05550
LNAT_RS06150
LNAT_RS08375
LNAT_RS02935
LNAT_RS02950
LNAT_RS01055
LNAT_RS05495
LNAT_RS02695
LNAT_RS07805
LNAT_RS08345
LNAT_RS05735
Caminibacter mediatlanticus TB-2
Histidine Kinase
Response Regulator
CMTB2_RS01565
CMTB2_RS02000
CMTB2_RS03330
CMTB2_RS04295
CMTB2_RS04540
CMTB2_RS08410
CMTB2_RS04945
CMTB2_RS02775
CMTB2_RS09120
CMTB2_RS09150
CMTB2_RS01560
CMTB2_RS00265
CMTB2_RS03325
CMTB2_RS04290
CMTB2_RS04535
CMTB2_RS08405
CMTB2_RS04940
CMTB2_RS02770
CMTB2_RS02785
CMTB2_RS06025
CMTB2_RS06445
CMTB2_RS06715
CMTB2_RS03650
CMTB2_RS01805
CMTB2_RS06475
Cetia pacifica TB6
Histidine Kinase
Response Regulator
C6V80_RS00585
C6V80_RS01550
C6V80_RS02095
C6V80_RS02540
C6V80_RS02700
C6V80_RS03205
C6V80_RS03250
C6V80_RS04780
C6V80_RS05615
C6V80_RS06725
C6V80_RS07300
C6V80_RS07635
C6V80_RS03125
C6V80_RS00580
C6V80_RS01555
C6V80_RS03620
C6V80_RS02545
C6V80_RS02705
C6V80_RS03200
C6V80_RS03245
C6V80_RS04775
C6V80_RS05610
C6V80_RS06730
C6V80_RS07305
C6V80_RS07290
C6V80_RS08085
C6V80_RS07845
C6V80_RS07835
C6V80_RS06510
C6V80_RS06430
C6V80_RS00610
C6V80_RS03860
C6V80_RS02285
Nautilia profundicola AmH
Histidine Kinase
Response Regulator
NAMH_RS01235
NAMH_RS01510
NAMH_RS01935
NAMH_RS02775
NAMH_RS04990
NAMH_RS06720
NAMH_RS08910
NAMH_RS09090
NAMH_RS06285
NAMH_RS08990
NAMH_RS06410
NAMH_RS00095
NAMH_RS01240
NAMH_RS01515
NAMH_RS01930
NAMH_RS02770
NAMH_RS04995
NAMH_RS03310
NAMH_RS00640
NAMH_RS03705
NAMH_RS06290
NAMH_RS06275
NAMH_RS01010
NAMH_RS01700
NAMH_RS05165
NAMH_RS02495
NAMH_RS07420
HisKA
HATPase_c
NIT
SBP
ABC_sub_bind
PAS_4
Hpt
REC
HTH
Sigma54
GGDEF
PAS
PAS_9
PAS_3
EAL

Hydrogenimonas thermophila EP1-55-1

Histidine Kinase | Response Regulator

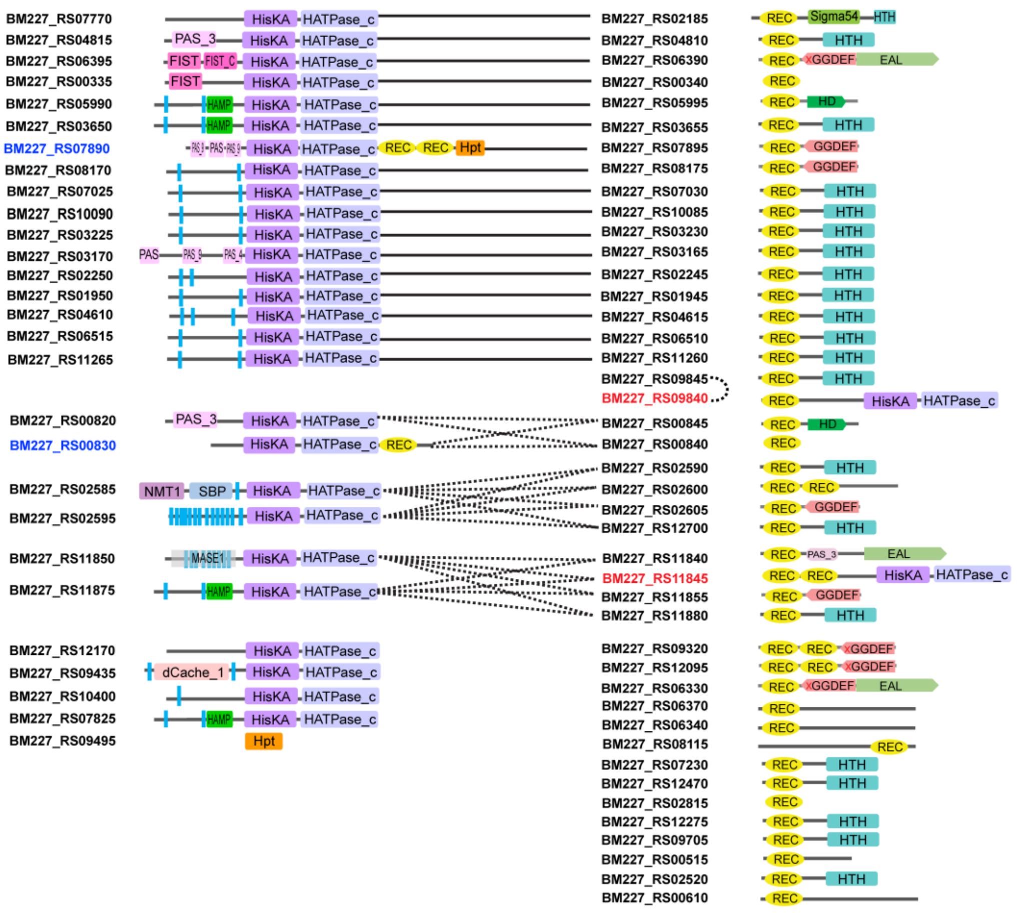

Nitratiruptor sp. SB155-2

Histidine Kinase | Response Regulator

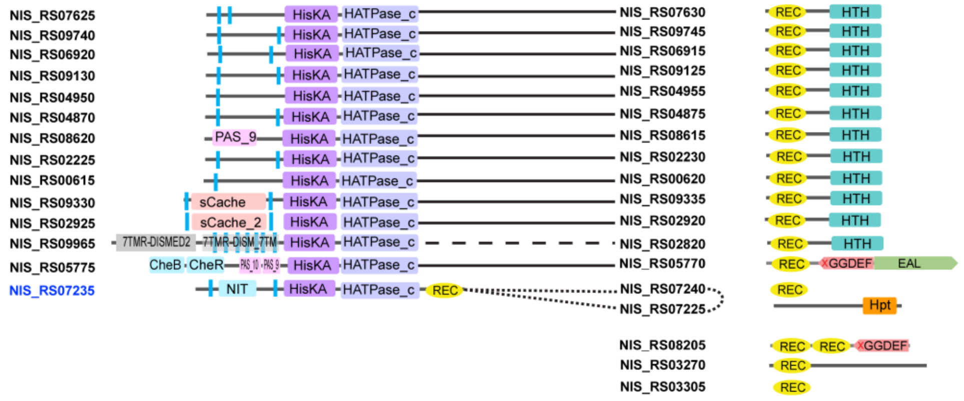

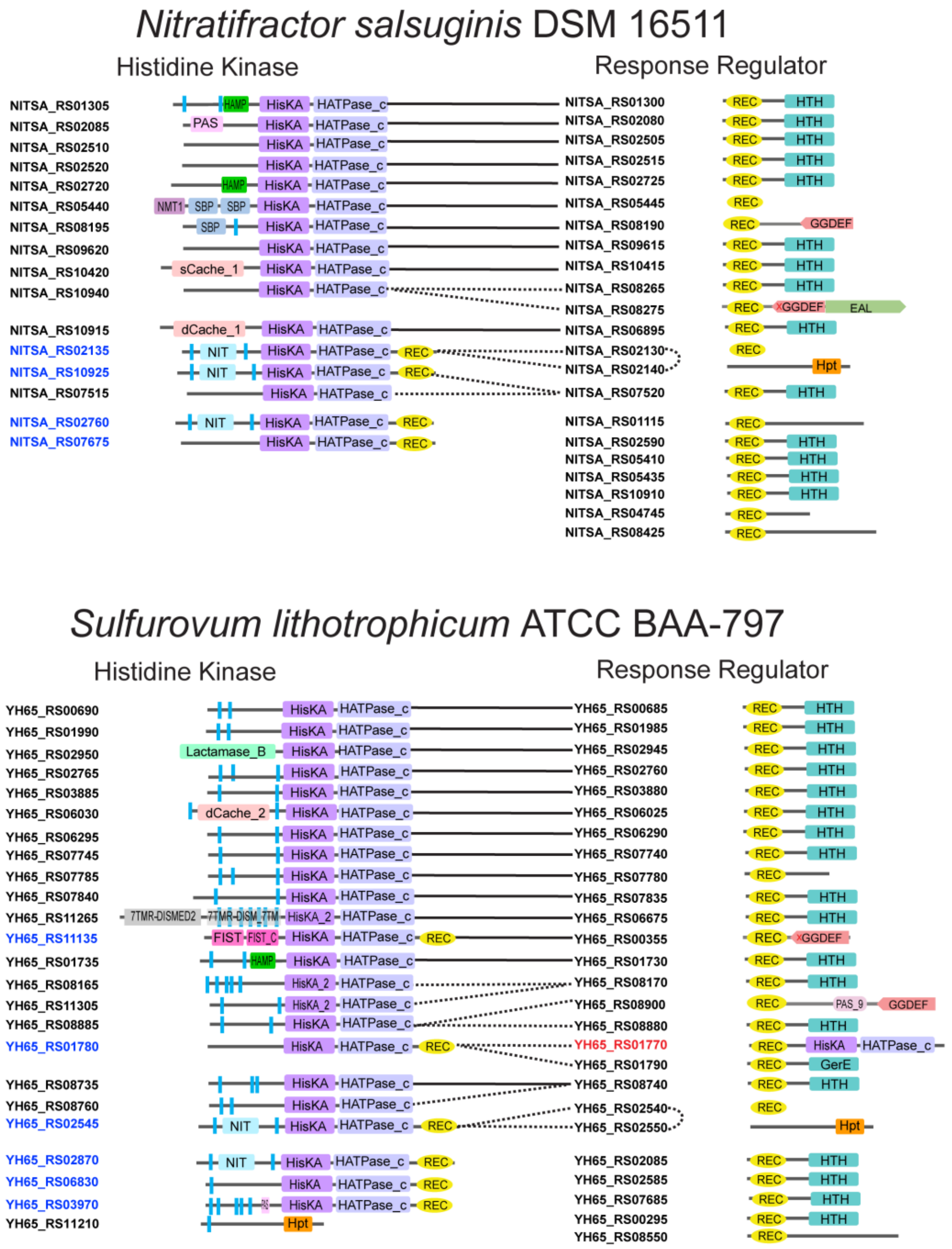

Nitratifractor salsuginis DSM 16511
Histidine Kinase
Response Regulator
NITSA_RS01305
NITSA_RS02085
NITSA_RS02510
NITSA_RS02520
NITSA_RS02720
NITSA_RS05440
NITSA_RS08195
NITSA_RS09620
NITSA_RS10420
NITSA_RS10940
NITSA_RS10915
NITSA_RS02135
NITSA_RS10925
NITSA_RS07515
NITSA_RS02760
NITSA_RS07675
NITSA_RS01300
NITSA_RS02080
NITSA_RS02505
NITSA_RS02515
NITSA_RS02725
NITSA_RS05445
NITSA_RS08190
NITSA_RS09615
NITSA_RS10415
NITSA_RS08265
NITSA_RS08275
NITSA_RS06895
NITSA_RS02130
NITSA_RS02140
NITSA_RS07520
NITSA_RS01115
NITSA_RS02590
NITSA_RS05410
NITSA_RS05435
NITSA_RS10910
NITSA_RS04745
NITSA_RS08425
HAMP
PAS
NMT1
SBP
sCache_1
dCache_1
NIT
HisKA
HATPase_c
REC
HTH
GGDEF
EAL
Hpt
Sulfurovum lithotrophicum ATCC BAA-797
Histidine Kinase
Response Regulator
YH65_RS00690
YH65_RS01990
YH65_RS02950
YH65_RS02765
YH65_RS03885
YH65_RS06030
YH65_RS06295
YH65_RS07745
YH65_RS07785
YH65_RS07840
YH65_RS11265
YH65_RS11135
YH65_RS01735
YH65_RS08165
YH65_RS11305
YH65_RS08885
YH65_RS01780
YH65_RS08735
YH65_RS08760
YH65_RS02545
YH65_RS02870
YH65_RS06830
YH65_RS03970
YH65_RS11210
YH65_RS00685
YH65_RS01985
YH65_RS02945
YH65_RS02760
YH65_RS03880
YH65_RS06025
YH65_RS06290
YH65_RS07740
YH65_RS07780
YH65_RS07835
YH65_RS06675
YH65_RS00355
YH65_RS01730
YH65_RS08170
YH65_RS08900
YH65_RS08880
YH65_RS01770
YH65_RS01790
YH65_RS08740
YH65_RS02540
YH65_RS02550
YH65_RS02085
YH65_RS02585
YH65_RS07685
YH65_RS00295
YH65_RS08550
Lactamase_B
dCache_2
7TMR-DISMED2
7TMR-DISM_7TM
HisKA_2
FIST
FIST_C
HAMP
PAS_9
GerE

Sulfurimonas gotlandica GD1

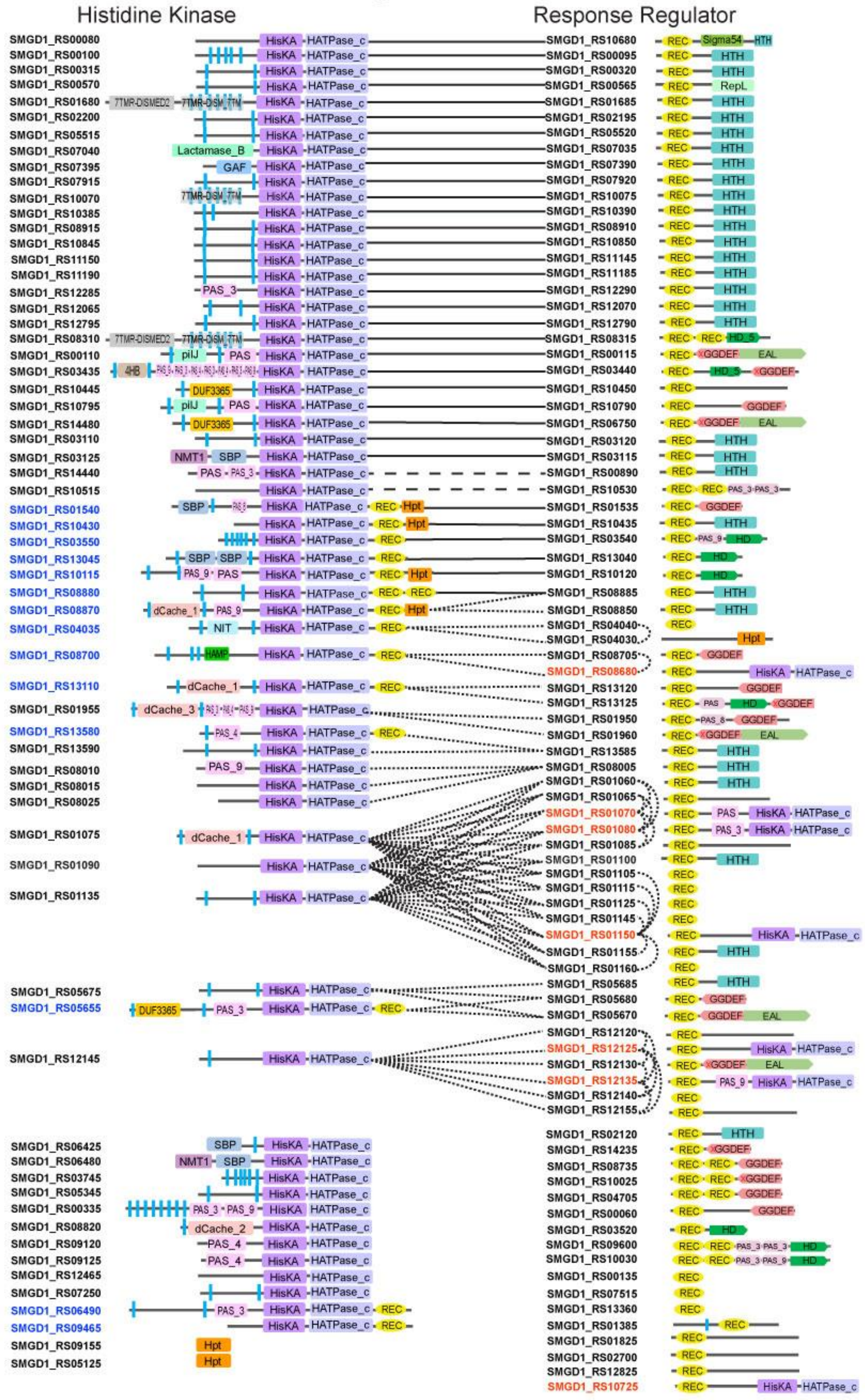

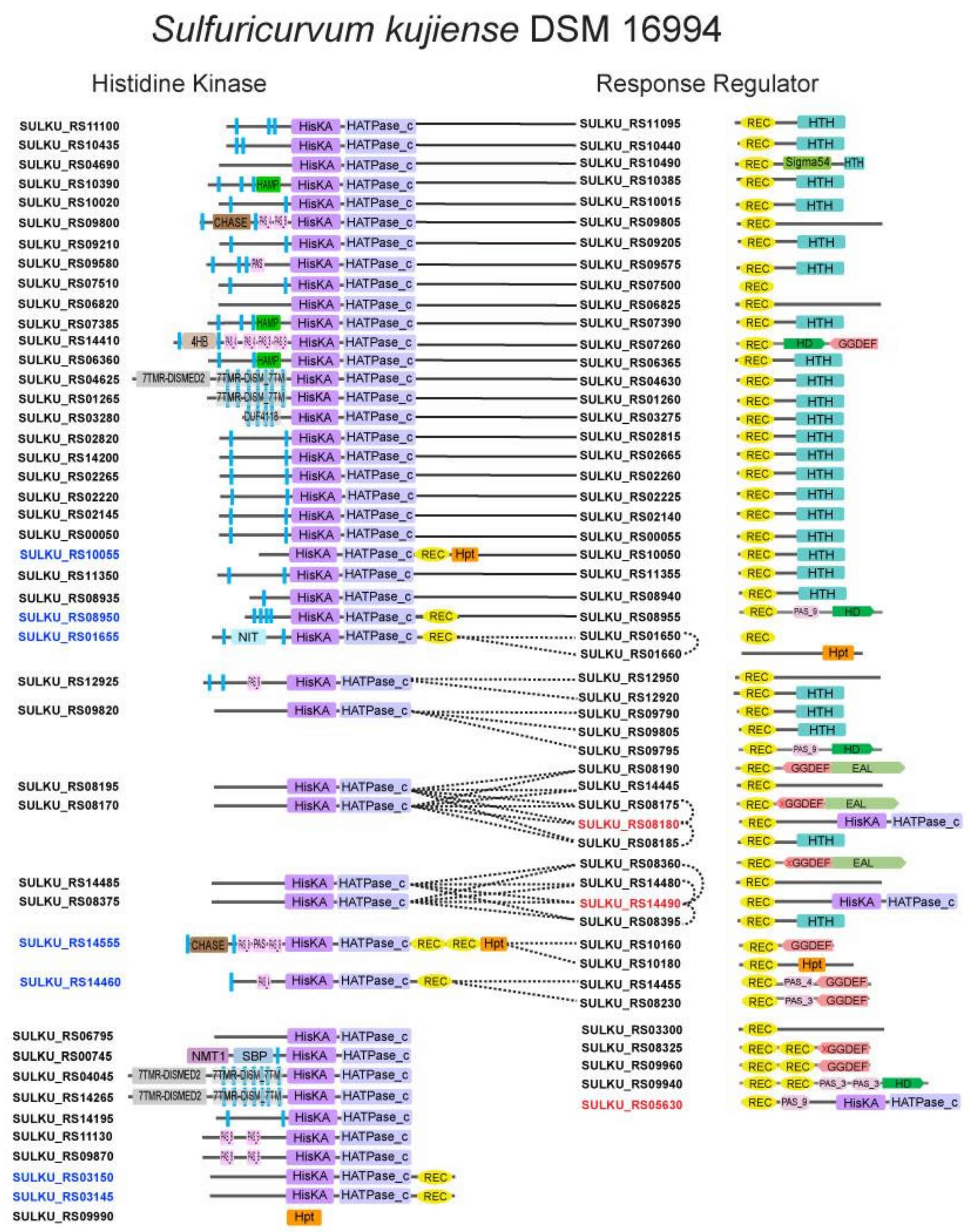

Sulfuricurvum kujiense DSM 16994
Histidine Kinase
Response Regulator
SULKU_RS11100
SULKU_RS10435
SULKU_RS04690
SULKU_RS10390
SULKU_RS10020
SULKU_RS09800
SULKU_RS09210
SULKU_RS09580
SULKU_RS07510
SULKU_RS06820
SULKU_RS07385
SULKU_RS14410
SULKU_RS06360
SULKU_RS04625
SULKU_RS01265
SULKU_RS03280
SULKU_RS02820
SULKU_RS14200
SULKU_RS02265
SULKU_RS02220
SULKU_RS02145
SULKU_RS00050
SULKU_RS10055
SULKU_RS11350
SULKU_RS08935
SULKU_RS08950
SULKU_RS01655
SULKU_RS12925
SULKU_RS09820
SULKU_RS08195
SULKU_RS08170
SULKU_RS14485
SULKU_RS08375
SULKU_RS14555
SULKU_RS14460
SULKU_RS06795
SULKU_RS00745
SULKU_RS04045
SULKU_RS14265
SULKU_RS14195
SULKU_RS11130
SULKU_RS09870
SULKU_RS03150
SULKU_RS03145
SULKU_RS09990
SULKU_RS11095
SULKU_RS10440
SULKU_RS10490
SULKU_RS10385
SULKU_RS10015
SULKU_RS09805
SULKU_RS09205
SULKU_RS09575
SULKU_RS07500
SULKU_RS06825
SULKU_RS07390
SULKU_RS07260
SULKU_RS06365
SULKU_RS04630
SULKU_RS01260
SULKU_RS03275
SULKU_RS02815
SULKU_RS02665
SULKU_RS02260
SULKU_RS02225
SULKU_RS02140
SULKU_RS00055
SULKU_RS10050
SULKU_RS11355
SULKU_RS08940
SULKU_RS08955
SULKU_RS01650
SULKU_RS01660
SULKU_RS12950
SULKU_RS12920
SULKU_RS09790
SULKU_RS09805
SULKU_RS09795
SULKU_RS08190
SULKU_RS14445
SULKU_RS08175
SULKU_RS08180
SULKU_RS08185
SULKU_RS08360
SULKU_RS14480
SULKU_RS14490
SULKU_RS08395
SULKU_RS10160
SULKU_RS10180
SULKU_RS14455
SULKU_RS08230
SULKU_RS03300
SULKU_RS08325
SULKU_RS09960
SULKU_RS09940
SULKU_RS05630
HisKA
HATPase_c
REC
HTH
Hpt
Sigma54
HAMP
CHASE
4HB
HD
GGDEF
EAL
7TMR-DISMED2
NIT
NMT1
SBP

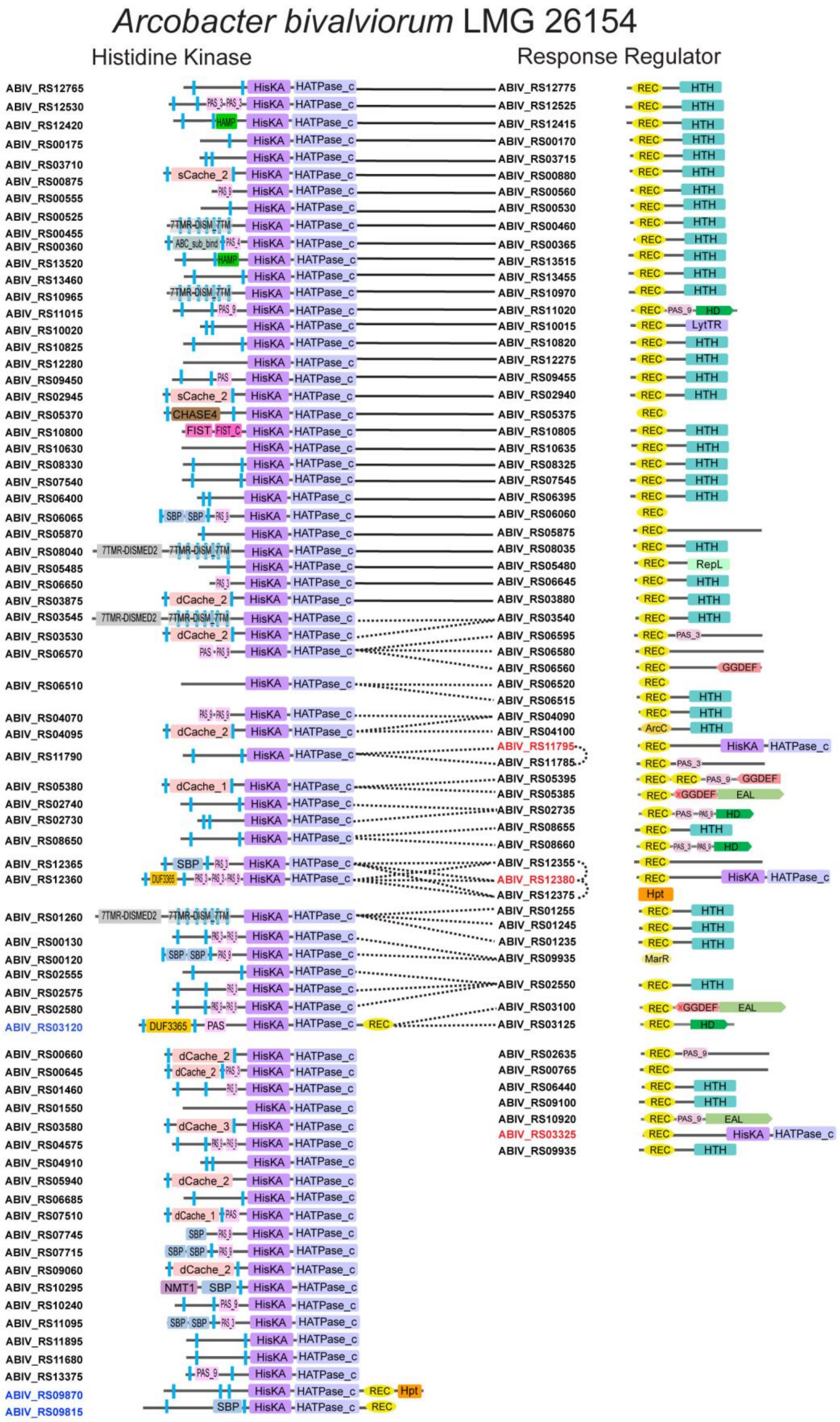
Arcobacter bivalviorum LMG 26154
Histidine Kinase
Response Regulator
ABIV_RS12765
ABIV_RS12530
ABIV_RS12420
ABIV_RS00175
ABIV_RS03710
ABIV_RS00875
ABIV_RS00555
ABIV_RS00525
ABIV_RS00455
ABIV_RS00360
ABIV_RS13520
ABIV_RS13460
ABIV_RS10965
ABIV_RS11015
ABIV_RS10020
ABIV_RS10825
ABIV_RS12280
ABIV_RS09450
ABIV_RS02945
ABIV_RS05370
ABIV_RS10800
ABIV_RS10630
ABIV_RS08330
ABIV_RS07540
ABIV_RS06400
ABIV_RS06065
ABIV_RS05870
ABIV_RS08040
ABIV_RS05485
ABIV_RS06650
ABIV_RS03875
ABIV_RS03545
ABIV_RS03530
ABIV_RS06570
ABIV_RS06510
ABIV_RS04070
ABIV_RS04095
ABIV_RS11790
ABIV_RS05380
ABIV_RS02740
ABIV_RS02730
ABIV_RS08650
ABIV_RS12365
ABIV_RS12360
ABIV_RS01260
ABIV_RS00130
ABIV_RS00120
ABIV_RS02555
ABIV_RS02575
ABIV_RS02580
ABIV_RS03120
ABIV_RS00660
ABIV_RS00645
ABIV_RS01460
ABIV_RS01550
ABIV_RS03580
ABIV_RS04575
ABIV_RS04910
ABIV_RS05940
ABIV_RS06685
ABIV_RS07510
ABIV_RS07745
ABIV_RS07715
ABIV_RS09060
ABIV_RS10295
ABIV_RS10240
ABIV_RS11095
ABIV_RS11895
ABIV_RS11680
ABIV_RS13375
ABIV_RS09870
ABIV_RS09815
ABIV_RS12775
ABIV_RS12525
ABIV_RS12415
ABIV_RS00170
ABIV_RS03715
ABIV_RS00880
ABIV_RS00560
ABIV_RS00530
ABIV_RS00460
ABIV_RS00365
ABIV_RS13515
ABIV_RS13455
ABIV_RS10970
ABIV_RS11020
ABIV_RS10015
ABIV_RS10820
ABIV_RS12275
ABIV_RS09455
ABIV_RS02940
ABIV_RS05375
ABIV_RS10805
ABIV_RS10635
ABIV_RS08325
ABIV_RS07545
ABIV_RS06395
ABIV_RS06060
ABIV_RS05875
ABIV_RS08035
ABIV_RS05480
ABIV_RS06645
ABIV_RS03880
ABIV_RS03540
ABIV_RS06595
ABIV_RS06580
ABIV_RS06560
ABIV_RS06520
ABIV_RS06515
ABIV_RS04090
ABIV_RS04100
ABIV_RS11795
ABIV_RS11785
ABIV_RS05395
ABIV_RS05385
ABIV_RS02735
ABIV_RS08655
ABIV_RS08660
ABIV_RS12355
ABIV_RS12380
ABIV_RS12375
ABIV_RS01255
ABIV_RS01245
ABIV_RS01235
ABIV_RS09935
ABIV_RS02550
ABIV_RS03100
ABIV_RS03125
ABIV_RS02635
ABIV_RS00765
ABIV_RS06440
ABIV_RS09100
ABIV_RS10920
ABIV_RS03325
ABIV_RS09935

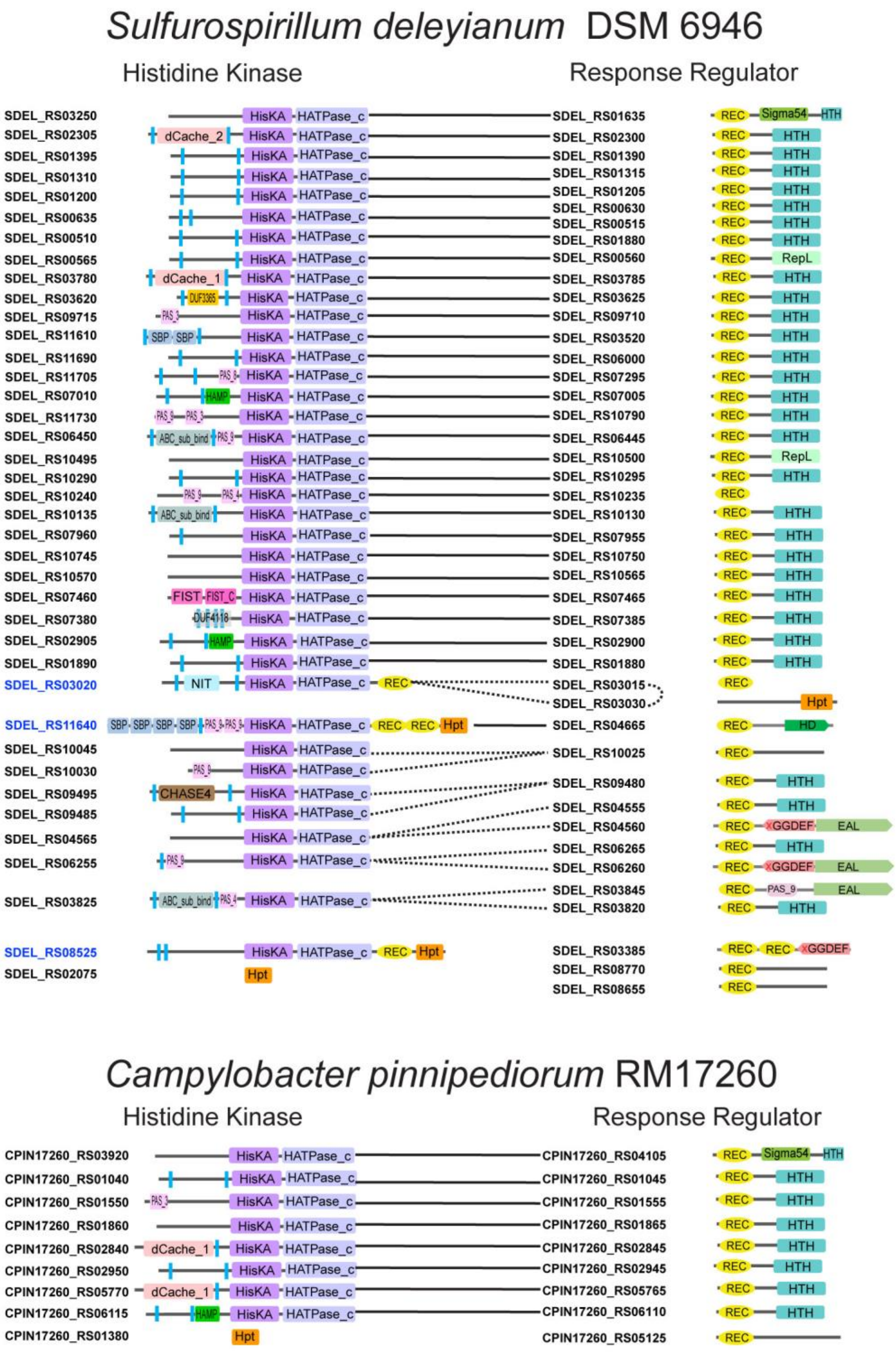
Sulfurospirillum deleyianum DSM 6946
Histidine Kinase
Response Regulator
SDEL_RS03250
SDEL_RS02305
SDEL_RS01395
SDEL_RS01310
SDEL_RS01200
SDEL_RS00635
SDEL_RS00510
SDEL_RS00565
SDEL_RS03780
SDEL_RS03620
SDEL_RS09715
SDEL_RS11610
SDEL_RS11690
SDEL_RS11705
SDEL_RS07010
SDEL_RS11730
SDEL_RS06450
SDEL_RS10495
SDEL_RS10290
SDEL_RS10240
SDEL_RS10135
SDEL_RS07960
SDEL_RS10745
SDEL_RS10570
SDEL_RS07460
SDEL_RS07380
SDEL_RS02905
SDEL_RS01890
SDEL_RS03020
SDEL_RS11640
SDEL_RS10045
SDEL_RS10030
SDEL_RS09495
SDEL_RS09485
SDEL_RS04565
SDEL_RS06255
SDEL_RS03825
SDEL_RS08525
SDEL_RS02075
SDEL_RS01635
SDEL_RS02300
SDEL_RS01390
SDEL_RS01315
SDEL_RS01205
SDEL_RS00630
SDEL_RS00515
SDEL_RS01880
SDEL_RS00560
SDEL_RS03785
SDEL_RS03625
SDEL_RS09710
SDEL_RS03520
SDEL_RS06000
SDEL_RS07295
SDEL_RS07005
SDEL_RS10790
SDEL_RS06445
SDEL_RS10500
SDEL_RS10295
SDEL_RS10235
SDEL_RS10130
SDEL_RS07955
SDEL_RS10750
SDEL_RS10565
SDEL_RS07465
SDEL_RS07385
SDEL_RS02900
SDEL_RS01880
SDEL_RS03015
SDEL_RS03030
SDEL_RS04665
SDEL_RS10025
SDEL_RS09480
SDEL_RS04555
SDEL_RS04560
SDEL_RS06265
SDEL_RS06260
SDEL_RS03845
SDEL_RS03820
SDEL_RS03385
SDEL_RS08770
SDEL_RS08655
HisKA
HATPase_c
dCache_2
dCache_1
DUF3365
PAS_3
SBP
PAS_9
HAMP
ABC_sub_bind
PAS_4
FIST
FIST_C
DUF4118
NIT
CHASE4
REC
Hpt
Sigma54
HTH
RepL
HD
GGDEF
EAL
Campylobacter pinnipediorum RM17260
Histidine Kinase
Response Regulator
CPIN17260_RS03920
CPIN17260_RS01040
CPIN17260_RS01550
CPIN17260_RS01860
CPIN17260_RS02840
CPIN17260_RS02950
CPIN17260_RS05770
CPIN17260_RS06115
CPIN17260_RS01380
CPIN17260_RS04105
CPIN17260_RS01045
CPIN17260_RS01555
CPIN17260_RS01865
CPIN17260_RS02845
CPIN17260_RS02945
CPIN17260_RS05765
CPIN17260_RS06110
CPIN17260_RS05125

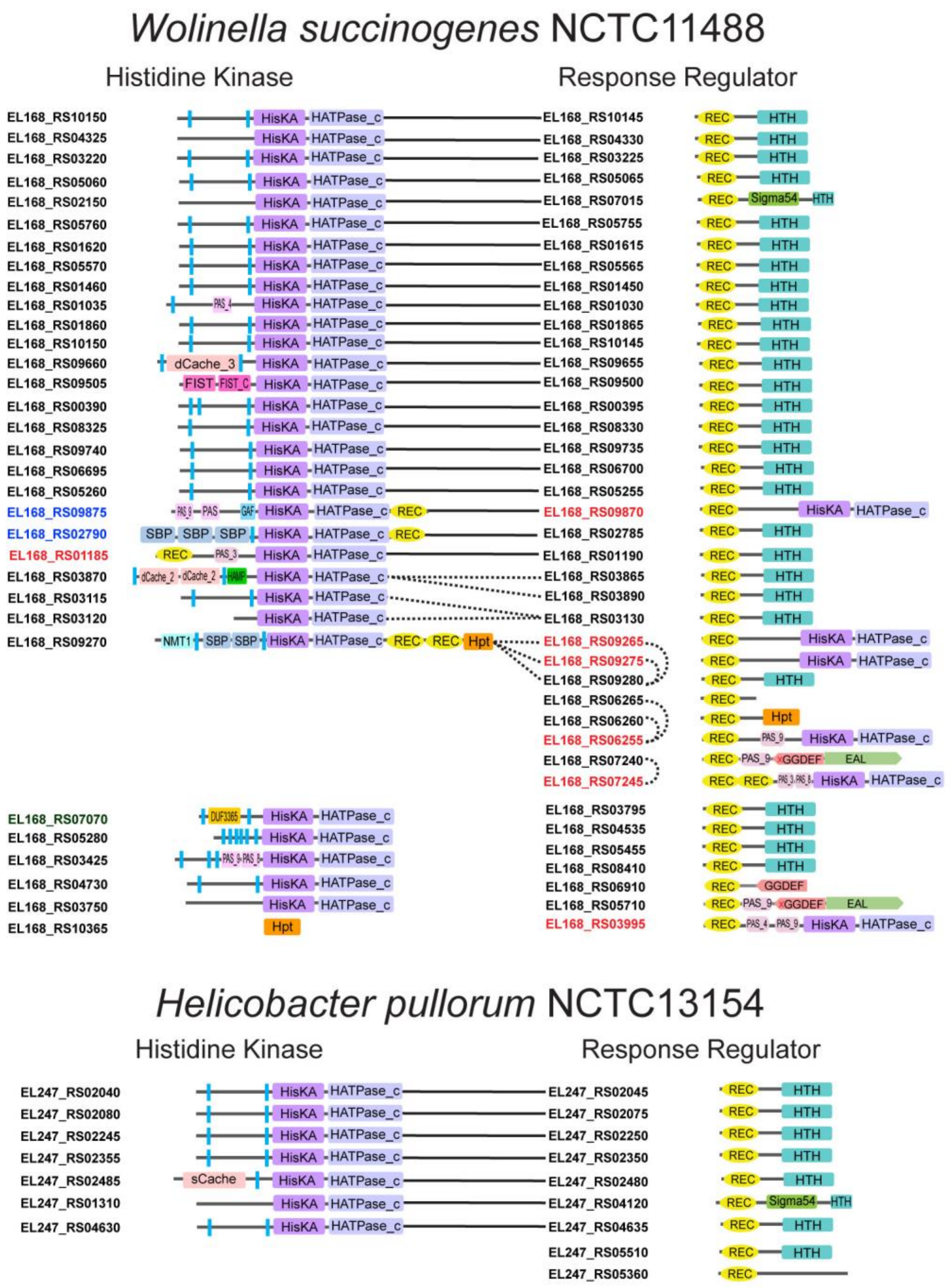

Fig. S3. Domain architectures of all HKs and RRs in representative species of each genus within the *Campylobacterota* phylum. HHKs are highlighted in blue and HRRs are highlighted in red. The solid line connects the adjacent HK and RR genes; dashed line represents potential 1:1 functional link of HK and RR gene pair within four genes distance; dotted line indicates potential functional links among multiple HK and RR genes within four genes distance.
