# Supplementary material for: Evolutionary Principles of Bacterial Signaling Capacity and Complexity
Source: mBio. 2022 May 10;13(3):e00764-22. doi: 10.1128/mbio.00764-22 (PMC9239204; doi:10.1128/mbio.00764-22)
Supplement: FIG S4 [file mbio.00764-22-sf004.pdf]

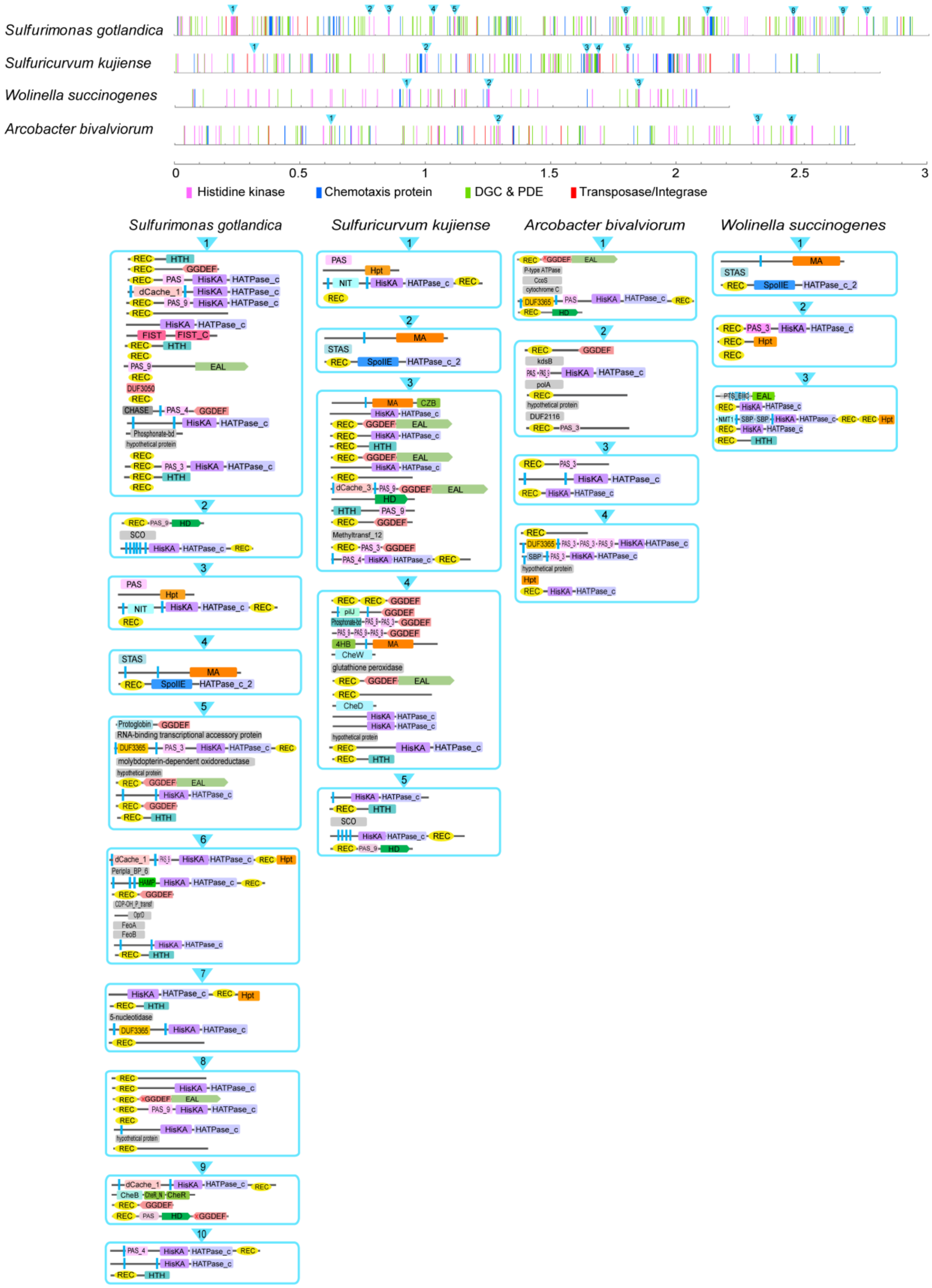

Fig. S4. Atypical HK enriched clusters in four *Campylobacterota* species with abundant TCS genes. Each genome is linearized and depicted as a scale line, and signal transduction genes are represented as colored lines based on their starting location. The HHK and HRR enriched regions are illustrated at the bottom panel.
