# Supplementary material for: Evolutionary Principles of Bacterial Signaling Capacity and Complexity
Source: mBio. 2022 May 10;13(3):e00764-22. doi: 10.1128/mbio.00764-22 (PMC9239204; doi:10.1128/mbio.00764-22)
Supplement: FIG S5 [file mbio.00764-22-sf005.pdf]

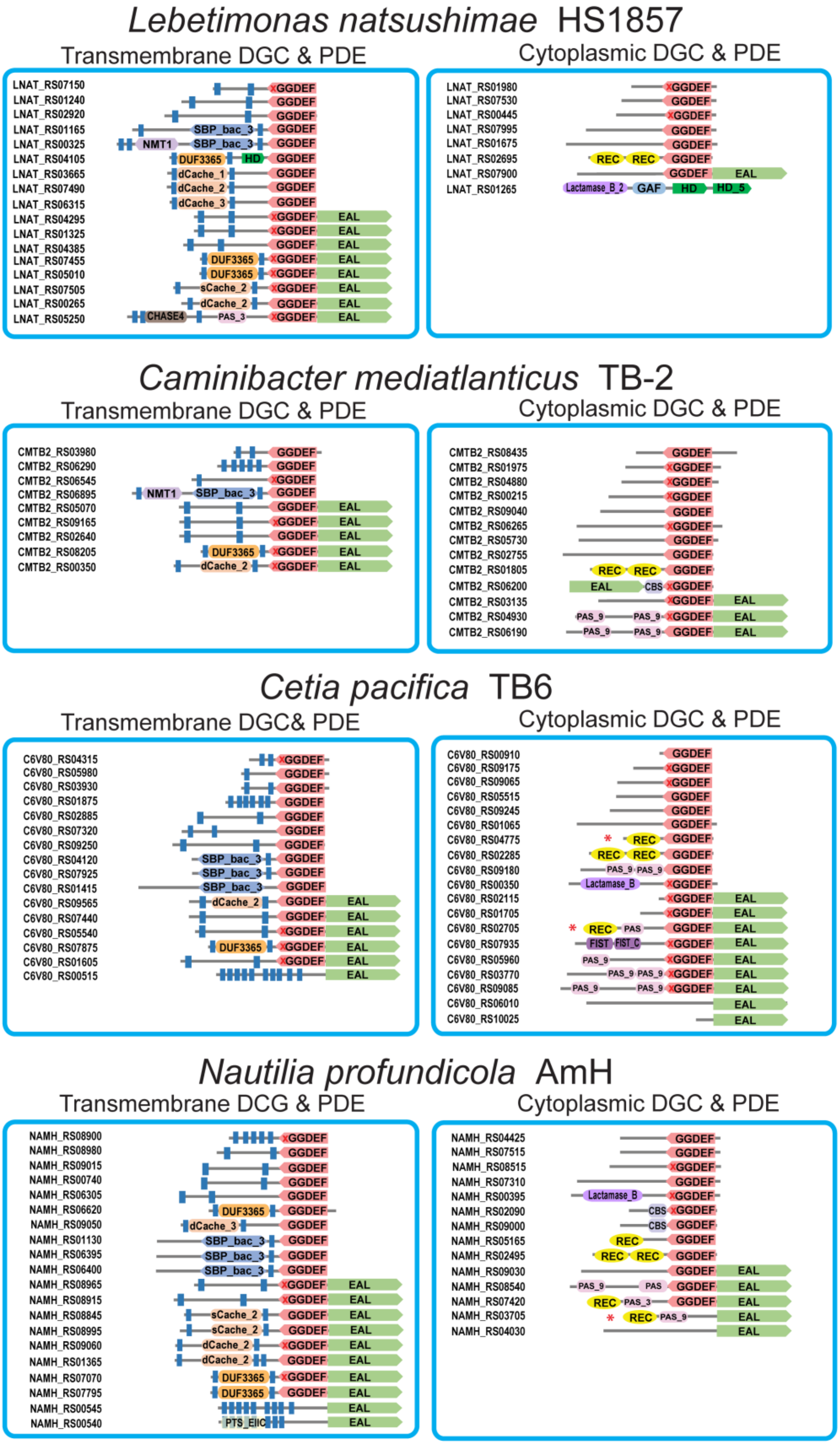
Lebetimonas natsushimae HS1857
Transmembrane DGC & PDE
LNAT_RS07150
LNAT_RS01240
LNAT_RS02920
LNAT_RS01165
LNAT_RS00325
LNAT_RS04105
LNAT_RS03665
LNAT_RS07490
LNAT_RS06315
LNAT_RS04295
LNAT_RS01325
LNAT_RS04385
LNAT_RS07455
LNAT_RS05010
LNAT_RS07505
LNAT_RS00265
LNAT_RS05250
Cytoplasmic DGC & PDE
LNAT_RS01980
LNAT_RS07530
LNAT_RS00445
LNAT_RS07995
LNAT_RS01675
LNAT_RS02695
LNAT_RS07900
LNAT_RS01265
Caminibacter mediatlanticus TB-2
Transmembrane DGC & PDE
CMTB2_RS03980
CMTB2_RS06290
CMTB2_RS06545
CMTB2_RS06895
CMTB2_RS05070
CMTB2_RS09165
CMTB2_RS02640
CMTB2_RS08205
CMTB2_RS00350
Cytoplasmic DGC & PDE
CMTB2_RS08435
CMTB2_RS01975
CMTB2_RS04880
CMTB2_RS00215
CMTB2_RS09040
CMTB2_RS06265
CMTB2_RS05730
CMTB2_RS02755
CMTB2_RS01805
CMTB2_RS06200
CMTB2_RS03135
CMTB2_RS04930
CMTB2_RS06190
Cetia pacifica TB6
Transmembrane DGC& PDE
C6V80_RS04315
C6V80_RS05980
C6V80_RS03930
C6V80_RS01875
C6V80_RS02885
C6V80_RS07320
C6V80_RS09250
C6V80_RS04120
C6V80_RS07925
C6V80_RS01415
C6V80_RS09565
C6V80_RS07440
C6V80_RS05540
C6V80_RS07875
C6V80_RS01605
C6V80_RS00515
Cytoplasmic DGC & PDE
C6V80_RS00910
C6V80_RS09175
C6V80_RS09065
C6V80_RS05515
C6V80_RS09245
C6V80_RS01065
C6V80_RS04775
C6V80_RS02285
C6V80_RS09180
C6V80_RS00350
C6V80_RS02115
C6V80_RS01705
C6V80_RS02705
C6V80_RS07935
C6V80_RS05960
C6V80_RS03770
C6V80_RS09085
C6V80_RS06010
C6V80_RS10025
Nautilia profundicola AmH
Transmembrane DCG & PDE
NAMH_RS08900
NAMH_RS08980
NAMH_RS09015
NAMH_RS00740
NAMH_RS06305
NAMH_RS06620
NAMH_RS09050
NAMH_RS01130
NAMH_RS06395
NAMH_RS06400
NAMH_RS08965
NAMH_RS08915
NAMH_RS08845
NAMH_RS08995
NAMH_RS09060
NAMH_RS01365
NAMH_RS07070
NAMH_RS07795
NAMH_RS00545
NAMH_RS00540
Cytoplasmic DGC & PDE
NAMH_RS04425
NAMH_RS07515
NAMH_RS08515
NAMH_RS07310
NAMH_RS00395
NAMH_RS02090
NAMH_RS09000
NAMH_RS05165
NAMH_RS02495
NAMH_RS09030
NAMH_RS08540
NAMH_RS07420
NAMH_RS03705
NAMH_RS04030

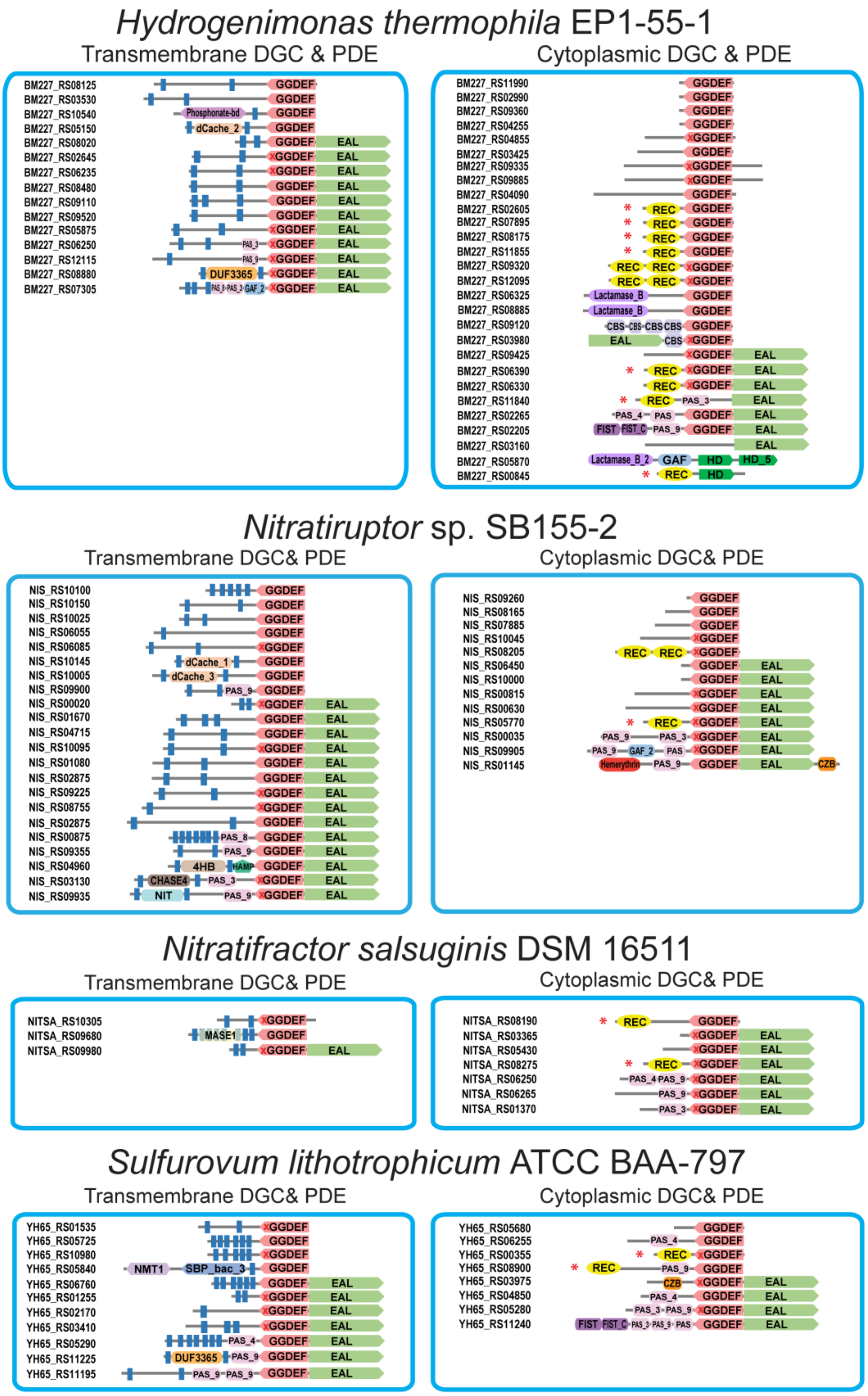

Hydrogenimonas thermophila EP1-55-1
Transmembrane DGC & PDE
BM227_RS08125
BM227_RS03530
BM227_RS10540
BM227_RS05150
BM227_RS08020
BM227_RS02645
BM227_RS06235
BM227_RS08480
BM227_RS09110
BM227_RS09520
BM227_RS05875
BM227_RS06250
BM227_RS12115
BM227_RS08880
BM227_RS07305
Cytoplasmic DGC & PDE
BM227_RS11990
BM227_RS02990
BM227_RS09360
BM227_RS04255
BM227_RS04855
BM227_RS03425
BM227_RS09335
BM227_RS09885
BM227_RS04090
BM227_RS02605
BM227_RS07895
BM227_RS08175
BM227_RS11855
BM227_RS09320
BM227_RS12095
BM227_RS06325
BM227_RS08885
BM227_RS09120
BM227_RS03980
BM227_RS09425
BM227_RS06390
BM227_RS06330
BM227_RS11840
BM227_RS02265
BM227_RS02205
BM227_RS03160
BM227_RS05870
BM227_RS00845
Phosphonate-bd
dCache_2
DUF3365
GGDEF
EAL
REC
Lactamase_B
CBS
PAS_3
PAS_4
PAS_9
FIST
FIST_C
GAF
HD
HD_5
Nitratiruptor sp. SB155-2
Transmembrane DGC& PDE
NIS_RS10100
NIS_RS10150
NIS_RS10025
NIS_RS06055
NIS_RS06085
NIS_RS10145
NIS_RS10005
NIS_RS09900
NIS_RS00020
NIS_RS01670
NIS_RS04715
NIS_RS10095
NIS_RS01080
NIS_RS02875
NIS_RS09225
NIS_RS08755
NIS_RS02875
NIS_RS00875
NIS_RS09355
NIS_RS04960
NIS_RS03130
NIS_RS09935
dCache_1
dCache_3
PAS_8
4HB
HAMP
CHASE4
NIT
Cytoplasmic DGC& PDE
NIS_RS09260
NIS_RS08165
NIS_RS07885
NIS_RS10045
NIS_RS08205
NIS_RS06450
NIS_RS10000
NIS_RS00815
NIS_RS00630
NIS_RS05770
NIS_RS00035
NIS_RS09905
NIS_RS01145
GAF_2
Hemerythrin
CZB
Nitratifractor salsuginis DSM 16511
Transmembrane DGC& PDE
NITSA_RS10305
NITSA_RS09680
NITSA_RS09980
MASE1
Cytoplasmic DGC& PDE
NITSA_RS08190
NITSA_RS03365
NITSA_RS05430
NITSA_RS08275
NITSA_RS06250
NITSA_RS06265
NITSA_RS01370
Sulfurovum lithotrophicum ATCC BAA-797
Transmembrane DGC& PDE
YH65_RS01535
YH65_RS05725
YH65_RS10980
YH65_RS05840
YH65_RS06760
YH65_RS01255
YH65_RS02170
YH65_RS03410
YH65_RS05290
YH65_RS11225
YH65_RS11195
NMT1
SBP_bac_3
Cytoplasmic DGC& PDE
YH65_RS05680
YH65_RS06255
YH65_RS00355
YH65_RS08900
YH65_RS03975
YH65_RS04850
YH65_RS05280
YH65_RS11240

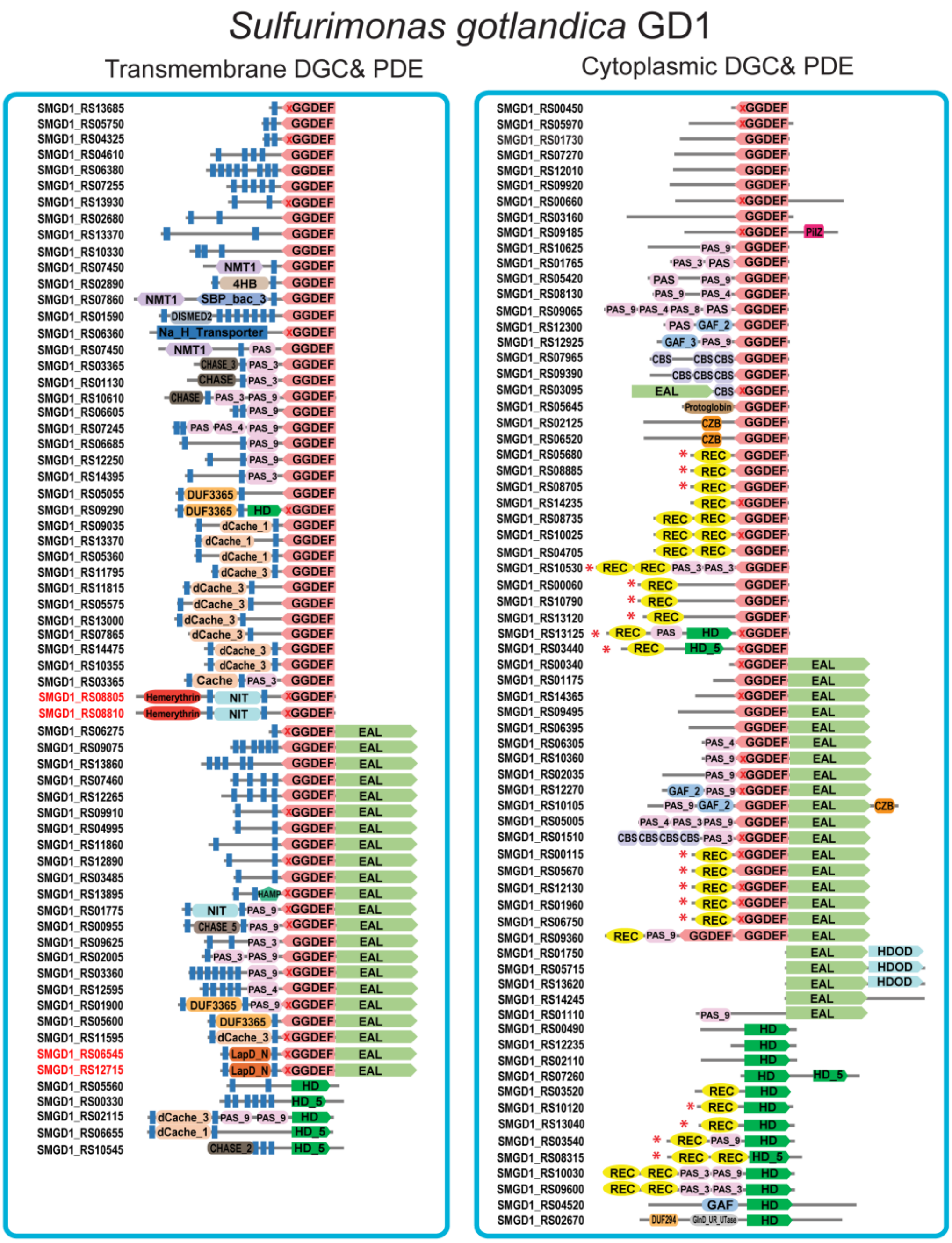

Sulfurimonas gotlandica GD1
Transmembrane DGC& PDE
SMGD1_RS13685
SMGD1_RS05750
SMGD1_RS04325
SMGD1_RS04610
SMGD1_RS06380
SMGD1_RS07255
SMGD1_RS13930
SMGD1_RS02680
SMGD1_RS13370
SMGD1_RS10330
SMGD1_RS07450
SMGD1_RS02890
SMGD1_RS07860
SMGD1_RS01590
SMGD1_RS06360
SMGD1_RS07450
SMGD1_RS03365
SMGD1_RS01130
SMGD1_RS10610
SMGD1_RS06605
SMGD1_RS07245
SMGD1_RS06685
SMGD1_RS12250
SMGD1_RS14395
SMGD1_RS05055
SMGD1_RS09290
SMGD1_RS09035
SMGD1_RS13370
SMGD1_RS05360
SMGD1_RS11795
SMGD1_RS11815
SMGD1_RS05575
SMGD1_RS13000
SMGD1_RS07865
SMGD1_RS14475
SMGD1_RS10355
SMGD1_RS03365
SMGD1_RS08805
SMGD1_RS08810
SMGD1_RS06275
SMGD1_RS09075
SMGD1_RS13860
SMGD1_RS07460
SMGD1_RS12265
SMGD1_RS09910
SMGD1_RS04995
SMGD1_RS11860
SMGD1_RS12890
SMGD1_RS03485
SMGD1_RS13895
SMGD1_RS01775
SMGD1_RS00955
SMGD1_RS09625
SMGD1_RS02005
SMGD1_RS03360
SMGD1_RS12595
SMGD1_RS01900
SMGD1_RS05600
SMGD1_RS11595
SMGD1_RS06545
SMGD1_RS12715
SMGD1_RS05560
SMGD1_RS00330
SMGD1_RS02115
SMGD1_RS06655
SMGD1_RS10545
Cytoplasmic DGC& PDE
SMGD1_RS00450
SMGD1_RS05970
SMGD1_RS01730
SMGD1_RS07270
SMGD1_RS12010
SMGD1_RS09920
SMGD1_RS00660
SMGD1_RS03160
SMGD1_RS09185
SMGD1_RS10625
SMGD1_RS01765
SMGD1_RS05420
SMGD1_RS08130
SMGD1_RS09065
SMGD1_RS12300
SMGD1_RS12925
SMGD1_RS07965
SMGD1_RS09390
SMGD1_RS03095
SMGD1_RS05645
SMGD1_RS02125
SMGD1_RS06520
SMGD1_RS05680
SMGD1_RS08885
SMGD1_RS08705
SMGD1_RS14235
SMGD1_RS08735
SMGD1_RS10025
SMGD1_RS04705
SMGD1_RS10530
SMGD1_RS00060
SMGD1_RS10790
SMGD1_RS13120
SMGD1_RS13125
SMGD1_RS03440
SMGD1_RS00340
SMGD1_RS01175
SMGD1_RS14365
SMGD1_RS09495
SMGD1_RS06395
SMGD1_RS06305
SMGD1_RS10360
SMGD1_RS02035
SMGD1_RS12270
SMGD1_RS10105
SMGD1_RS05005
SMGD1_RS01510
SMGD1_RS00115
SMGD1_RS05670
SMGD1_RS12130
SMGD1_RS01960
SMGD1_RS06750
SMGD1_RS09360
SMGD1_RS01750
SMGD1_RS05715
SMGD1_RS13620
SMGD1_RS14245
SMGD1_RS01110
SMGD1_RS00490
SMGD1_RS12235
SMGD1_RS02110
SMGD1_RS07260
SMGD1_RS03520
SMGD1_RS10120
SMGD1_RS13040
SMGD1_RS03540
SMGD1_RS08315
SMGD1_RS10030
SMGD1_RS09600
SMGD1_RS04520
SMGD1_RS02670

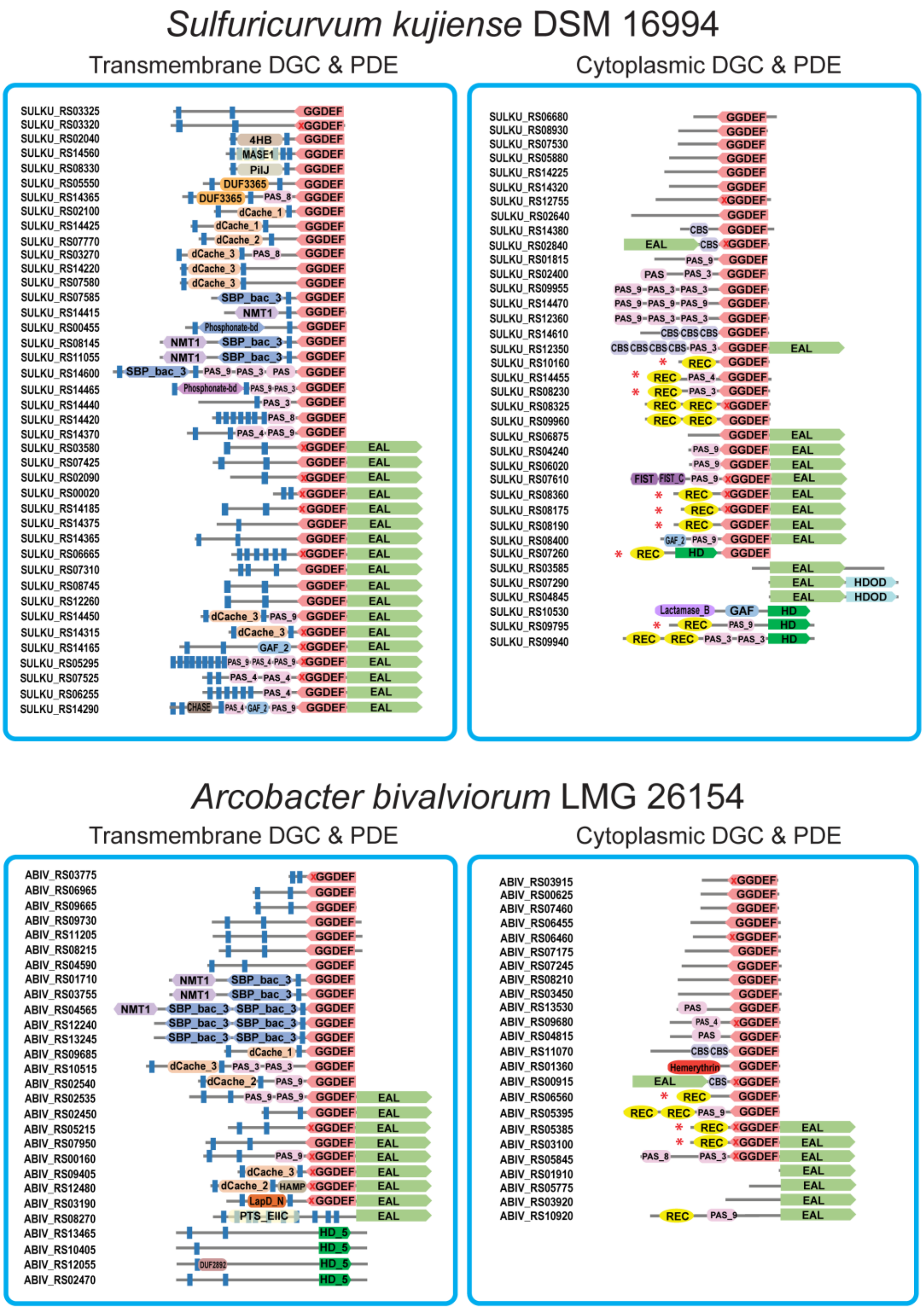

Sulfuricurvum kujiense DSM 16994
Transmembrane DGC & PDE
Cytoplasmic DGC & PDE
SULKU_RS03325
SULKU_RS03320
SULKU_RS02040
SULKU_RS14560
SULKU_RS08330
SULKU_RS05550
SULKU_RS14365
SULKU_RS02100
SULKU_RS14425
SULKU_RS07770
SULKU_RS03270
SULKU_RS14220
SULKU_RS07580
SULKU_RS07585
SULKU_RS14415
SULKU_RS00455
SULKU_RS08145
SULKU_RS11055
SULKU_RS14600
SULKU_RS14465
SULKU_RS14440
SULKU_RS14420
SULKU_RS14370
SULKU_RS03580
SULKU_RS07425
SULKU_RS02090
SULKU_RS00020
SULKU_RS14185
SULKU_RS14375
SULKU_RS14365
SULKU_RS06665
SULKU_RS07310
SULKU_RS08745
SULKU_RS12260
SULKU_RS14450
SULKU_RS14315
SULKU_RS14165
SULKU_RS05295
SULKU_RS07525
SULKU_RS06255
SULKU_RS14290
SULKU_RS06680
SULKU_RS08930
SULKU_RS07530
SULKU_RS05880
SULKU_RS14225
SULKU_RS14320
SULKU_RS12755
SULKU_RS02640
SULKU_RS14380
SULKU_RS02840
SULKU_RS01815
SULKU_RS02400
SULKU_RS09955
SULKU_RS14470
SULKU_RS12360
SULKU_RS14610
SULKU_RS12350
SULKU_RS10160
SULKU_RS14455
SULKU_RS08230
SULKU_RS08325
SULKU_RS09960
SULKU_RS06875
SULKU_RS04240
SULKU_RS06020
SULKU_RS07610
SULKU_RS08360
SULKU_RS08175
SULKU_RS08190
SULKU_RS08400
SULKU_RS07260
SULKU_RS03585
SULKU_RS07290
SULKU_RS04845
SULKU_RS10530
SULKU_RS09795
SULKU_RS09940
GGDEF
EAL
HDOD
HD
REC
PAS
CBS
GAF
Arcobacter bivalviorum LMG 26154
Transmembrane DGC & PDE
Cytoplasmic DGC & PDE
ABIV_RS03775
ABIV_RS06965
ABIV_RS09665
ABIV_RS09730
ABIV_RS11205
ABIV_RS08215
ABIV_RS04590
ABIV_RS01710
ABIV_RS03755
ABIV_RS04565
ABIV_RS12240
ABIV_RS13245
ABIV_RS09685
ABIV_RS10515
ABIV_RS02540
ABIV_RS02535
ABIV_RS02450
ABIV_RS05215
ABIV_RS07950
ABIV_RS00160
ABIV_RS09405
ABIV_RS12480
ABIV_RS03190
ABIV_RS08270
ABIV_RS13465
ABIV_RS10405
ABIV_RS12055
ABIV_RS02470
ABIV_RS03915
ABIV_RS00625
ABIV_RS07460
ABIV_RS06455
ABIV_RS06460
ABIV_RS07175
ABIV_RS07245
ABIV_RS08210
ABIV_RS03450
ABIV_RS13530
ABIV_RS09680
ABIV_RS04815
ABIV_RS11070
ABIV_RS01360
ABIV_RS00915
ABIV_RS06560
ABIV_RS05395
ABIV_RS05385
ABIV_RS03100
ABIV_RS05845
ABIV_RS01910
ABIV_RS05775
ABIV_RS03920
ABIV_RS10920

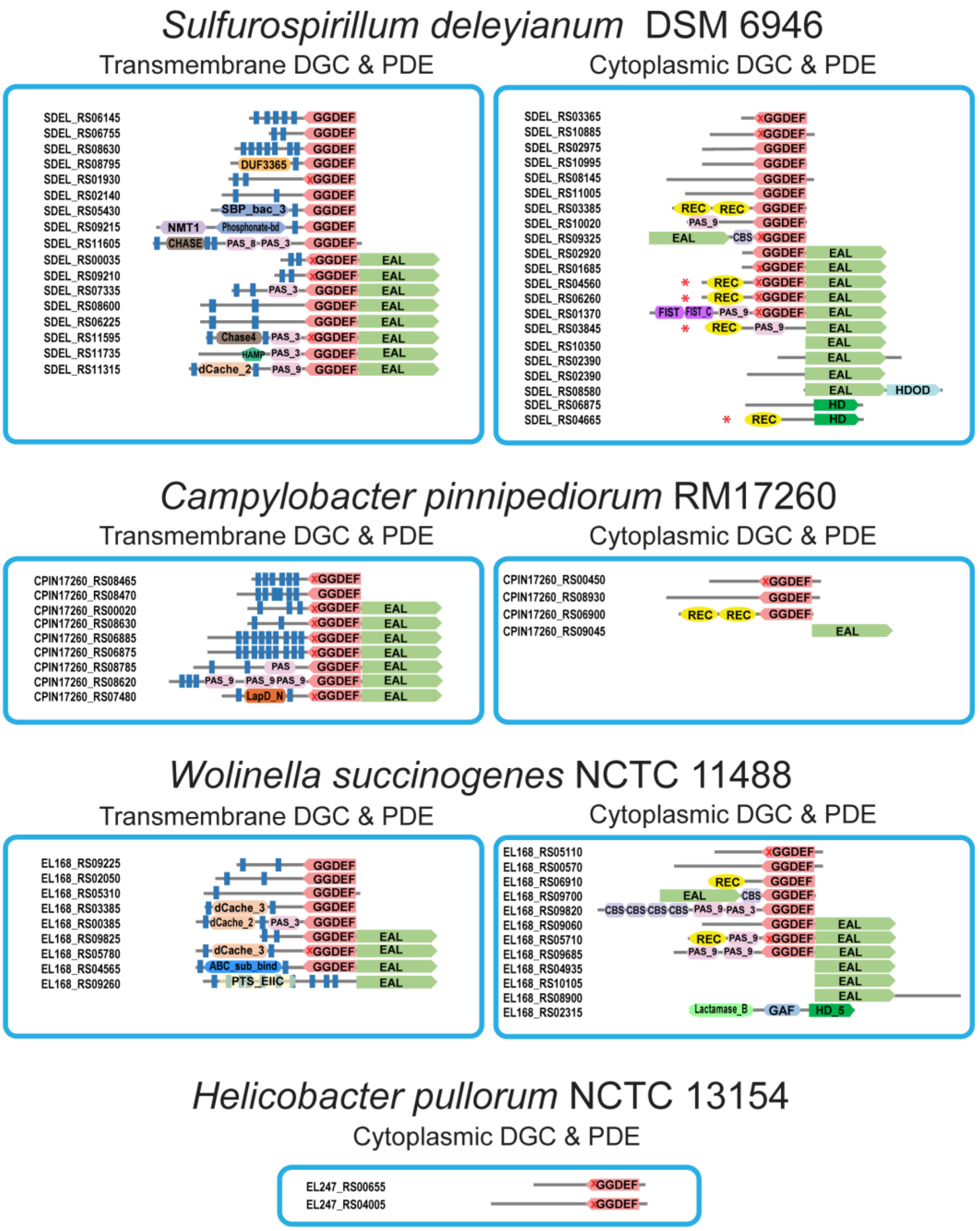

Fig. S5. Domain architectures of all DGCs and PDEs in representative species of each genus within the *Campylobacterota* phylum. These proteins are categorized as transmembrane and cytoplasmic proteins. The red asterisk represents a DGC or PDE gene with a REC domain, also in close proximity of a HK gene. The red cross in the GGDEF domain implies an enzymatically inactive DGC. In the box of *Sulfurimonas gotlandica* GD1, two pairs of DGCs are highlighted in red indicating recent gene duplication of two genes.
