# Supplementary figures and images for: Evolutionary Principles of Bacterial Signaling Capacity and Complexity
Source: mBio. 2022 May 10;13(3):e00764-22. doi: 10.1128/mbio.00764-22 (PMC9239204; doi:10.1128/mbio.00764-22)

A

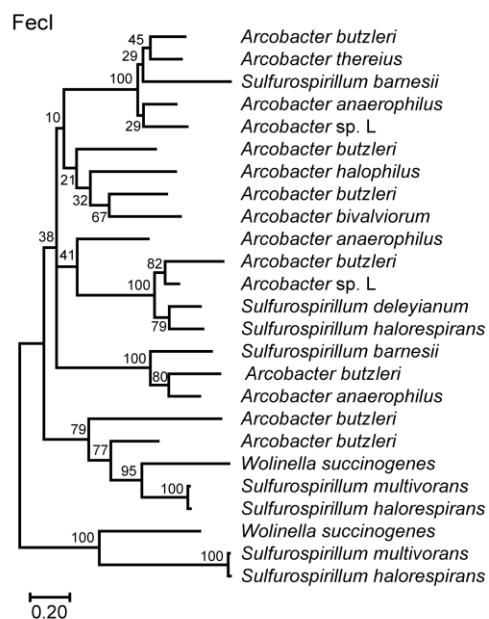

B

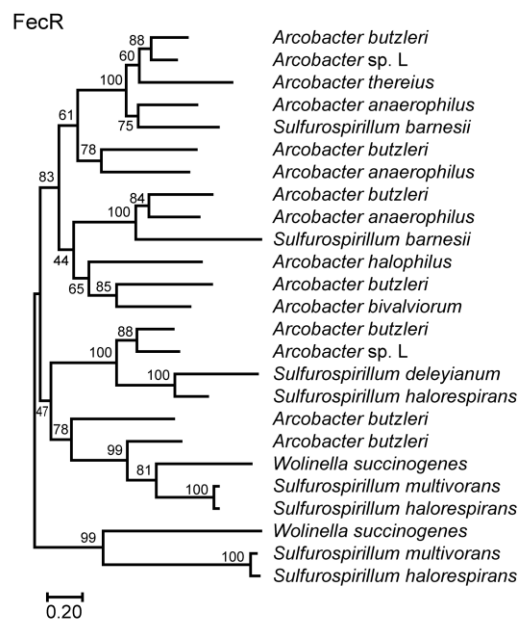

**Fig. S6.** Phylogenetic tree of FecI (a) and FecR (b) proteins in the *Campylobacterota* phylum.

Supplement: FIG S6 [file mbio.00764-22-sf006.pdf]
