# Supplementary material for: Evolutionary Principles of Bacterial Signaling Capacity and Complexity
Source: mBio. 2022 May 10;13(3):e00764-22. doi: 10.1128/mbio.00764-22 (PMC9239204; doi:10.1128/mbio.00764-22)
Supplement: FIG S7 [file mbio.00764-22-sf007.pdf]

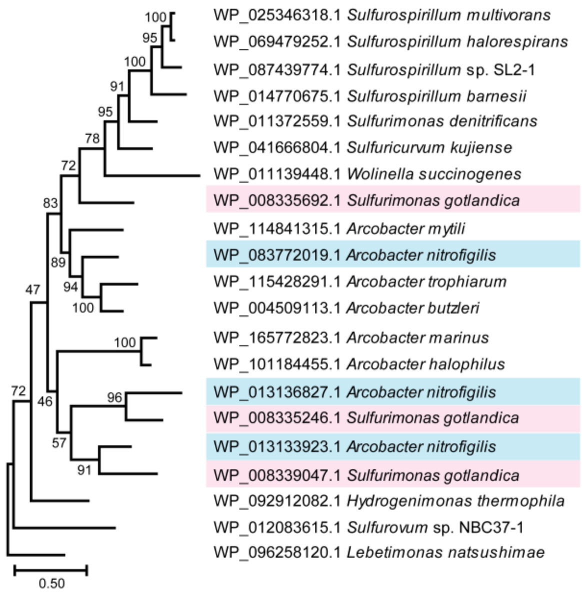

Fig. S7. Phylogenetic tree of adenylate cyclase in the *Campylobacterota* phylum. Pink, 3 adenylate cyclase homologs in *Sulfurimonas gotlandica* GD1; Blue, 3 adenylate cyclase homologs in *Arcobacter nitrofigilis* DSM 7299.
