# Supplementary material for: Evolutionary Principles of Bacterial Signaling Capacity and Complexity
Source: mBio. 2022 May 10;13(3):e00764-22. doi: 10.1128/mbio.00764-22 (PMC9239204; doi:10.1128/mbio.00764-22)
Supplement: FIG S8 [file mbio.00764-22-sf008.pdf]

QS Molecule	QS synthetase			QS Recceptor			
	Protein Name	PI assession	Domain	Protein Name	PI assession	Domain	Signal transduction system
**AI-2**	LuxS	WP_113597815.1	LuxS	LuxP	WP_113623853.1	Peripla_BP_4	
				LsrB	WP_000172465.1	Peripla_BP_4	
				RbsB	WP_005567919.1	Peripla_BP_4	
				LuxQ	WP_113623854.1	LuxQ, HisKA, HATPase_c, REC	TCS
				Tsr	WP_000919536.1	TarH, HAMP, MA	Chemosensory system
				PctA	WP_003148125.1	dCache_1, HAMP, MA	Chemosensory system
				TlpQ	WP_034017148.1	dCache_1, HAMP, MA	Chemosensory system
**AI-3**	LuxS ?	WP_001130215.1	LuxS	QseC	WP_000673362.1	2CSK_N, HisKA, HATPase_c	TCS
				QseB	WP_001221502.1	REC, HTH	TCS
				QseE	WP_001301750.1	HisKA, HATPase_c	TCS
				QseF	WP_001295369.1	REC, AAA	TCS
**AHL**	LuxI	WP_047863343.1	Acetyltransf_5	LuxR	WP_011263745.1	Autoind_bind, HTH LUXR	OCS
**CAI-1**	CqsA	WP_113598123.1	Aminotran_1_2	CqsS	WP_181710640.1	HisKA, HATPase_c, REC	TCS
**PQS**	PqsA	WP_003112552.1	AMP-binding, AMP-binding_C	PqsR	WP_003108614.1	HTH_1, LysR_substrate	TCS
	PqsB	WP_003108611.1	ACP_syn_III				
	PqsC	WP_003108612.1	ACP_syn_III, ACP_syn_III_C				
	PqsD	WP_003112550.1	ACP_syn_III, ACP_syn_III_C				
	PqsH	WP_003090354.1	FAD_binding_3				
**DSF**	RpfF	WP_011037027.1	ECH_1	RpfR	WP_006488779.1	FI, PAS, GGDEF, EAL	DGC & PDE
				RpfC	WP_011037026.1	HisKA, HATPase_c, REC, HPT	TCS
				RpfG	WP_011037024.1	REC, HD_5	TCS

Fig. S8. Domain analyses of proteins in QS pathways. QS pathways are sorted by QS molecules; reported synthetases and receptors are listed. The ligand binding domains for QS molecules in the receptors are highlighted in orange. All proteins listed in the table are from non-*Campylobacterota* species that have been experimentally studied for the specific QS pathway.
